# Influence of Vegetation Structure on Lemur Recolonization of Post‐Fire Habitats in Northwestern Madagascar

**DOI:** 10.1002/ajp.70186

**Published:** 2026-07-05

**Authors:** Naina Ratsimba Rabemananjara, Misa Rasolozaka, Marie Odile Ravolanirina, Rogula Marivola, Seheno Harilala Randriamiarantsoa, Romule Rakotondravony, Hanta Razafindraibe, Dominik Schüßler, Ute Radespiel

**Affiliations:** ^1^ Institute of Zoology University of Veterinary Medicine Hannover Hannover Germany; ^2^ Mention Sciences de la Vie et de l'Environnement, Faculté des Sciences, de Technologies et de l'Environnement University of Mahajanga Mahajanga Madagascar; ^3^ Mention of Zoology and Animal Biodiversity, Faculté des Sciences University of Antananarivo Antananarivo Madagascar; ^4^ Institute of Biology and Chemistry University of Hildesheim Hildesheim Germany

**Keywords:** Ankarafantsika, ecological requirements, fire ecology, lemur species richness, vegetation structure, woody species richness

## Abstract

Habitat quality is a key determinant of wildlife distribution. Fire degrades forests by increasing canopy openness and eliminating even large trees, providing shelter and necessary resources for many species. In Madagascar, fires are prominent, yet the mechanisms linking fire disturbance to lemur habitat occupation remain poorly understood. We conducted a study inside Ankarafantsika National Park during the dry seasons of 2022 and 2023, collecting data along paired burnt–unburnt transects in 18 sites containing burnt partitions with a 1–35‐year fire age. Using Generalized Linear Mixed Models, we assessed the effects of forest humidity, temperature, vegetation structure, and woody species richness on lemur occurrence, and evaluated the impact of fire parameters on significant abiotic and vegetation parameters. Lemur responses varied by body size and ecological specialization. *Eulemur fulvus* most likely occurred in humid habitats with high floristic diversity. Floristic diversity also explained best the presence of *Avahi occidentalis* and *Cheirogaleus medius*, while *Lepilemur edwardsi* was rather connected to dense multi‐layered canopies. Small‐bodied *Microcebus* species displayed structural and floristic tolerance. Woody species richness declined in the burnt forest and was highest in the unburnt forest. Fires impacted vegetation structure, increasing openness and understory density, while old burnt and unburnt forests maintained complex vertical layering. Woody species richness emerged as the strongest predictor of lemur species richness. These results indicate that fire primarily affects lemurs through floristic and structural pathways, emphasizing the need to preserve intact forest refugia to sustain lemur diversity under increasing fire pressure in dry forest landscapes of Madagascar.

## Summary

- Forest fires alter lemur habitats by reducing woody species richness and mature tree density.
- *Eulemur fulvus*, *Avahi occidentalis*, and *Cheirogaleus medius* preferentially occupied floristically diverse forests, suggesting the relevance of a diverse spectrum of food resources for species of divergent body size.
- The medium‐sized *Lepilemur edwardsi* was sensitive to fire‐induced loss of canopy integrity, limiting their persistence in burnt forests, while small‐bodied mouse lemurs (*Microcebus ravelobensis and Microcebus murinus*) exhibited high resilience to fire‐modulated habitat changes.
- Lemur species richness was strongly linked to woody species richness, underscoring the role of floristic richness in post‐fire lemur and habitat recovery.

## Graphical abstract

Lemur species respond differently to fire‐related vegetation changes (structure, species diversity) according to body size and ecological specialization in the dry deciduous forests of northwestern Madagascar.

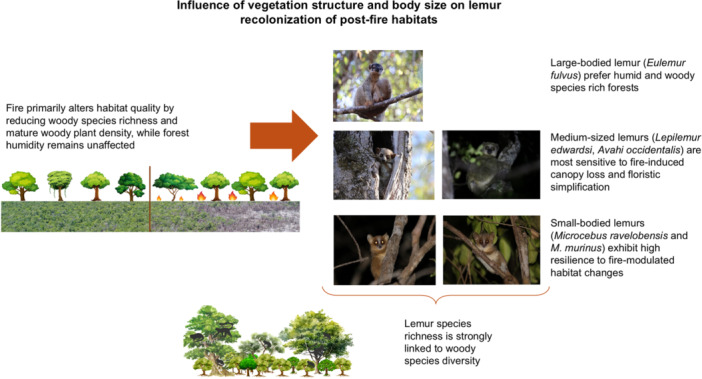

## 1 Introduction

Habitat complexity is a key driver of wildlife distribution, persistence, and biodiversity, as structural features such as canopy cover, tree density, understory complexity, and heterogeneity determine the availability of resources, shelter, and movement opportunities (Flesch 2017; Ishii et al. 2004; LaRue et al. 2019). Anthropogenic disturbances such as logging, fragmentation, and fire reduce habitat structural complexity, causing declines in canopy cover, tree density, and vertical stratification, with cascading negative consequences for arboreal wildlife diversity, abundance, and tree species richness (Lindenmayer et al. 2017; Estrada and Garber 2022). Among the wildlife groups most strongly affected by such structural habitat changes, primates stand out as particularly vulnerable, given their strict dependence on forest structural complexity for movement, foraging, shelter, and predator avoidance (Cant 1992; Estrada and Garber 2022). As predominantly arboreal mammals, primates rely on canopy height and connectivity to enable safe movement and support the diverse microhabitat requirements of multi‐species communities (Cudney‐Valenzuela et al. 2021, 2022). The sensitivity of primates to structural habitat changes, however, varies considerably among species. Large species with large home ranges are generally considered most sensitive to structural forest loss and fragmentation, as they depend on extensive areas of intact canopy to meet their energetic and spatial requirements (Symington 1988; Mitani et al. 2000). Smaller flexible species more frequently persist in structurally degraded landscapes, as they can exploit a wider range of microhabitats and food resources available in simplified forest structures (Bicca‐Marques 2003; Digby et al. 2011). For example, Spider monkeys (*Ateles* spp.), large and predominantly frugivorous primates, are considered particularly sensitive to disturbances due to their high spatial and resource requirements (Symington 1988), while howler monkeys (*Alouatta* spp.) are often comparatively more tolerant, capable of shifting their diet to leaves and secondary vegetation, using small home ranges, and adopting energy‐efficient activity patterns (Bicca‐Marques 2003). At the other end of the size spectrum, the smaller common marmosets (*Callithrix jacchus*) frequently occur in secondary forest and edge habitats (Digby et al. 2011). However, body size alone is a limited predictor of disturbance tolerance, as dietary specialization, locomotor requirements, shelter use, and behavioral flexibility all play important mediating roles (Estrada et al. 2017; Eppley et al. 2022). Notably, small body size does not universally confer resilience. For example, the pygmy tarsier (*Tarsius pumilus*) of Sulawesi, Indonesia, despite being one of the world's smallest primates, is a strict insectivore and montane forest specialist with a highly restricted distribution, and its populations are considered highly vulnerable to habitat loss and forest degradation driven by human encroachment (Grow et al. 2013).

Fire is one of the most pervasive ecological forces shaping tropical landscapes. While natural fires occur regularly in savannahs, grasslands, and Mediterranean‐climate regions (Keeley et al. 2011), they have been historically rare in humid tropical forests due to high moisture levels (Bond and Keeley 2005). Human activities have increased fire frequency and intensity across tropical regions (Cochrane 2003), removing biomass, opening the canopy, and altering microclimates in ways that favor fire‐tolerant vegetation over mature forest species (Fernandes‐Carvalho‐De‐Andrade et al. 2021; Moser et al. 2010). Moreover, when fires are repeated and severe, these effects accumulate over time, progressively shifting ecosystems toward grass‐dominated states and ultimately determining whether ecosystems can recover or cross tipping points toward permanently degraded states from which return is unlikely (Cochrane and Laurance 2002; González et al. 2022).

Fire can impact forest fauna through changes in habitat structure and resource availability (Lyon et al. 2012). Among the wildlife groups directly affected by such changes, arboreal primates are particularly sensitive, as fire‐driven reduction in canopy cover, tree density, and vertical stratification can directly compromise their movement, foraging, and predator avoidance strategies (Herzog et al. 2020; Lappan et al. 2020; Widyastuti et al. 2022). The nature of this response, however, varies depending on the ecology and locomotor habits of the species concerned. In savannah‐dwelling primates, fire‐driven openings in the canopy can initially appear beneficial by increasing predator visibility at greater distances; for example, studies on vervet monkeys (*Chlorocebus aethiops*) have shown that reduced ground cover in burnt areas alters ranging behavior and predation risk perception, with individuals adjusting their spatial use of burnt habitats accordingly (Jaffe and Isbell 2009). In strictly arboreal primates, however, the consequences of fire‐driven structural change appear to be mostly negative. Long‐tailed macaques (*Macaca fascicularis*) in Kutai National Park, for instance, responded to fire‐induced loss of tree diversity and canopy connectivity by dispersing widely and increasing their use of midstory and terrestrial travel routes, substantially increasing both predation risk and energetic costs (Berenstain 1986; Johnsingh 1986).

Madagascar is one of the world's most distinctive biodiversity hotspots, characterized by exceptional endemism with more than 90% of its fauna and flora being found nowhere else on Earth (Goodman and Benstead 2005). Lemurs, the endemic primate clade of Madagascar, comprise more than 100 species that vary remarkably in body size, ecology, and life history strategies (Mittermeier et al. 2023). Lemurs inhabit nearly all forested habitats from humid rainforests to dry deciduous and spiny forests, but this diversity is increasingly threatened by deforestation, fragmentation, and fires (Mittermeier et al. 2023). Fire is a dominant ecological and anthropogenic force in Madagascar, shaping vegetation and now contributing substantially to forest loss (Phelps et al. 2022). Although some grassy biomes have long been maintained by natural fire regimes (Solofondranohatra et al. 2020), most contemporary fires are human‐induced, associated with shifting cultivation (tavy), pasture creation, and hunting (Kull 2004). Recent satellite data revealed widespread fire anomalies across the island, challenging global assumptions about degradation dynamics (Joseph et al. 2023). The western dry deciduous forests are especially fire‐prone due to seasonal drought and flammable vegetation, making them one of the most threatened ecosystems worldwide (Nolan et al. 2020; Phelps et al. 2022). Malagasy forests have already undergone major changes in vegetation structure and composition, including increased abundance of grasses, dry‐adapted taxa, and deciduous trees, since human arrival on the island, particularly from 1900 AD onwards (Razanatsoa et al. 2022). In general, recurrent fires can reduce canopy height, increase gap frequency, and promote fire‐tolerant shrubs and grass. Over time, this process impedes regeneration and can permanently convert forests into degraded shrublands or grasslands with diminished capacity to support native fauna (Allerton et al. 2025; Harrison et al. 2024; Pereira et al. 2024). Despite these potential threats, the ecological consequences of fires on Madagascar's fauna remain poorly understood.

Ankarafantsika National Park (ANP) in northwestern Madagascar represents a key remnant of dry deciduous forest and an ideal setting to study fire–fauna interactions (Alonso and Hannah 2002). The National Park's history of recurrent wildfires (Rasolozaka et al. 2025) has produced a mosaic of forest patches at varying successional stages adjacent to intact primary forest (Goodman et al. 2021). This landscape offers a comparative framework to study lemur communities across a gradient of disturbance. The park is a key refugium for eight lemur species that differ in daily activity pattern, body mass, diet, and IUCN conservation status (Table S1), including two Critically Endangered species, the Coquerel's sifaka (*Propithecus coquereli*) and the Mongoose lemur (*Eulemur mongoz*) (Schwitzer et al. 2013), alongside the Common brown lemur (*Eulemur fulvus*), the Milne‐Edwards' sportive lemur (*Lepilemur edwardsi*), the Western woolly lemur (*Avahi occidentalis*), the Western fat‐tailed dwarf lemur (*Cheirogaleus medius*), the Gray mouse lemur (*Microcebus murinus*), and the Golden‐brown mouse lemur (*Microcebus ravelobensis*) (Radespiel and Razafindramanana 2013).

A prior study in ANP established that lemur species richness is highest in unburnt forests, with large‐bodied species that are dietary and locomotor specialists being most vulnerable to fire disturbance, while small‐bodied dietary generalists with flexible microhabitat use displayed greater resilience (Rabemananjara et al. 2025). Dietary specialists, such as the folivorous *P. coquereli* and *A. occidentalis*, may depend on a diverse array of plant species to meet their nutritional requirements and digest plant secondary compounds (Thalmann 2001; McGoogan 2011), while locomotor specialists such as *L. edwardsi* rely on specific canopy substrates for their characteristic clinging‐and‐leaping locomotion. In contrast, small‐bodied dietary generalists such as *M. murinus* and *M. ravelobensis* exploit a broad range of food resources and forest strata, and display considerable flexibility in shelter use, being capable of utilizing a wide variety of sleeping sites, including tree cavities, leaf nests, and dense vegetation tangles across structurally diverse habitats (Radespiel et al. 2003; Thorén et al. 2011), conferring greater overall flexibility in fire‐modified landscapes. While this suggests that dietary specialization, locomotor ecology, shelter use flexibility, and body size may jointly mediate species‐specific sensitivity to fire, the mechanisms underlying these patterns remain poorly understood. In particular, it is unclear how fire‐induced changes in vegetation composition and microhabitat availability translate into differential habitat use across species.

The present study addresses this hypothesis by examining how variations in vegetation structure and abiotic conditions shape the presence of an entire suite of lemur species in post‐fire habitats in northwestern Madagascar. Understanding these relationships is crucial for predicting ecosystem recovery trajectories and developing evidence‐based conservation strategies for fire‐prone landscapes. Specifically, we pursue two objectives: (1) evaluate whether variations in vegetation structure and abiotic conditions affect the presence of large‐, medium‐, and small‐bodied lemur species, and (2) assess whether these structural and abiotic elements differ depending on the underlying fire history. Based on ecological theory, empirical evidence from other tropical regions, and preliminary observations from Madagascar, we propose the following two hypotheses:

Lemur presence will be positively associated with greater structural complexity (e.g., canopy cover, tree species diversity, vertical stratification) and undisturbed abiotic conditions, with large‐bodied species showing the strongest dependence on intact canopy connectivity and structural heterogeneity, and small‐bodied, generalist species being most tolerant of fire‐modulated changes.

Vegetation parameters that influence lemur presence are impacted by the fire status (burnt/unburnt), fire severity, fire frequency, and the time that has passed since the last fire. Thus, fire‐related structural and floristic changes can explain species‐specific vulnerabilities of lemurs to fires at least partially.

## 2 Methods

### 2.1 Ethics Statement

This study followed the American Society of Primatologists' Principles for the Ethical Treatment of Non‐Human Primates. All fieldwork protocols were reviewed and approved by the Institute of Zoology, University of Veterinary Medicine Hannover, Germany. The research complied with the legal regulations of Madagascar and received authorization from the Ministère de l'Environnement et du Développement Durable (MEDD), the Direction Régionale de l'Environnement (DREDD) Boeny Betsiboka, and Madagascar National Parks (permit numbers: № 275/22/MEDD/SG/DGGE/DAPRNE/SCBE.Re, № 070/23/MEDD/SG/DGGE/DAPRNE/SCBE.Re, and № 263/23/MEDD/SG/DGGE/DAPRNE/SCB E.Re).

### 2.2 Study Sites and Ecological Context

Our study was conducted in ANP, the largest remaining protected area of dry deciduous forest in northwestern Madagascar (−16°08′60″ S, 46°57′0″ E), covering 1350 km^2^ (Figure S1). The park's topography includes river valleys at ~50 m above sea level (a.s.l.), slopes, and a calcitic plateau that rises southward to ~350 m a.s.l., forming escarpments in the eastern and southern areas (Alonso and Hannah 2002). Vegetation consists of seasonally dry forest with a mosaic of dry deciduous forests, dry thickets at higher elevations, moist riverine forests around lakes and upstream valleys, and *Raphia* swamp forests in downstream valleys (Goodman et al. 2021). Forest cover occurs across plateaus, slopes, and valleys. Rainfall averages 1000–1500 mm annually, concentrated between November and April, while the dry season from May to October is almost rainless (Alonso and Hannah 2002). ANP contains a rich and diverse flora with over 287 species of woody plants, of which 92% are endemic to Madagascar (Alonso and Hannah 2002).

This study was conducted in the same 18 study sites (Figure S1) that were already used in a previous study, representing different fire histories within ANP (Rasolozaka et al. 2025). Briefly, Landsat imagery (30 × 30 m resolution) from 1988 to 2023 was used to classify pixels as burnt or unburnt for each year based on the Normalized Burn Ratio (NBR) by subtracting pre‐ and post‐fire NBR values (dNBR; Key and Benson 2006) to infer the 35‐year fire history at each site. Fire severity was quantified with the same resolution for all sites and years using the dNBR (Keeley 2009). Sites were selected to contrast long‐unburnt areas with adjacent burnt areas that differed in fire frequency and time since the last fire, ensuring a cross‐section design while controlling for ecological context (Rasolozaka et al. 2025). After fieldwork, remote sensing data were re‐analyzed alongside field evidence to ground‐truth the burn status. Ground truthing consisted of (1) examining charcoal particles in soil samples taken from the surface down to 10 cm below the surface, (2) identifying fire scars on large tree trunks, and (3) surveying the transect for aboveground macro‐charcoal particles (Rasolozaka et al. 2025). The reconstruction of site‐specific fire history revealed that some previously classified unburnt areas had burned (sites 5, 4, 13, 2, 12, and 15) and that several sites had heterogeneous fire histories, with different transect partitions having burned in different years. Transect partitions therefore differed in time since the last fire (1 to > 35 years), fire frequency (1–7 events over 35 years), minimum fire intervals (1–31 years), and maximum burn severity (Table S2). Consequently, transects were subdivided into partitions based on their distinct fire histories. Fieldwork was conducted between September and November 2022 (late dry to early rainy season) and between May and November 2023 (dry season). No data was collected during the peak of the rainy season.

### 2.3 Collection of Vegetation and Abiotic Data

**Figure 1:**
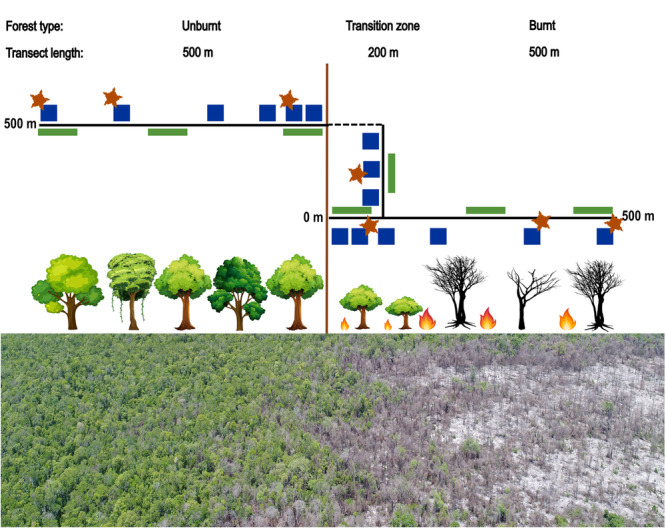
Standardized transect design for the field work. One 500 m‐burnt transect (perpendicular to the fire edge) is paired with one 500 m‐unburnt transect (perpendicular to the fire edge), both being connected by a 200 m long transition transect (parallel to the fire edge, 50 m inside the burnt forest). Green narrow rectangles represent floristic plots, blue squares indicate structural vegetation plots, and red stars show locations of abiotic data loggers. The solid black line marks transect lines, and the dashed line indicates a connecting path. Below: Drone image taken a few months after the 2021 fire near the research station of Ampijoroa in ANP, illustrating the spatial setting of the transects regarding the fire border. Photograph taken by Hiroki Sato.

At each site, we established a 1.2 km transect flagged and GPS‐mapped at 10 m intervals (Garmin GPSMAP 64). This transect consisted of two 500 m sections extending perpendicularly from the fire edge into burnt and unburnt forest, with the unburnt line offset by an additional 200 m section placed parallel to the fire edge but 50 m inside the burnt zone, capturing the transition between forest types (Figure 1). Data collection was conducted in all three transect sections, but for subsequent analyses, the transition zone was classified together with the burnt forest, as it represents an area that has been exposed to fire influences, and no statistical differences to the burnt forest could be detected (see below, Table S11).

Seven rectangular plots (3 m × 50 m; green rectangles in Figure 1) were placed along the 1.2 km transect at each site to capture the floristic richness across habitats with different fire exposure, with three plots located in unburnt forest and four in burnt forest, including one within the transition zone close to the fire edge where burnt and unburnt vegetation meet (Figure 1). In both the unburnt and burnt transects, plots were positioned using the same layout at distances of 5–55 m, 225–275 m, and 450–500 m from the fire edge and along the transect, while the transition plot was established at 75–125 m. Within each plot, we recorded all trees with a diameter at breast height (DBH) > 7 cm, identified them to species level whenever possible, and recorded and identified all shrubs taller than 1 m, to cover all mature specimens, if possible. For each plot, we thereby quantified woody plant density as the number of individual mature shrubs and trees and woody species richness as the total number of distinct species (range: 4–27 species per plot, Table S3).

In addition, 15 structural vegetation plots (5 m × 5 m, blue squares in Figure 1) were established along each transect. While six plots were located in the unburnt, nine were placed in the burnt forest, which includes three plots positioned in the transition zone (Figure 1). Plots were installed at fixed distances from the fire–forest edge (5, 10, 50, 100, 300, and 500 m) along burnt and unburnt transects and in the transition zone (50, 100, and 150 m). We counted all trees within each plot and categorized them into DBH classes (small: 0.25–5.0 cm; medium: 5.1–10.0 cm; large: > 10 cm) and height classes (small: 2.5–5.0 m; medium: 5.1–7.5 m; tall: 7.51–10.0 m; very tall: > 10 m). Finally, forest stratum cover was estimated in these plots for the lower (2.5–5.0 m), medium (5.1–10.0 m), and upper stratum (> 10 m) by using a modified Londo scale approach with 5% intervals (Londo 1976). As statistical analyses were performed at the floristic plot level and the vegetation plots located at 5, 10, and 50 m from the fire–forest edge were in close proximity to one another, the values used in the analyses corresponded to the mean DBH, tree height, and canopy cover calculated across these three plots. In all other cases, the vegetation plot with the highest locality fit for the floristic plot was used for data analyses.

Relative humidity (range: 40.75%–98.56%, Table S2) and maximum temperature (range: 28°C–44.6°C, Table S2) were measured hourly during each study period (10–13 days per site) using automated data loggers (GSP‐6, Elitech) placed along transects, and values were extracted for each day at 7 a.m. Three data loggers were located in the unburnt forest (at 5, 300, and 500 m), three in the burnt forest (at 5, 300, and 500 m), and one in the transition zone (at 100 m) (Figure 1). For each floristic plot within a site, we extracted abiotic data from the data logger located closest to it. Daily humidity and maximum temperature values were averaged across all available days to obtain a single representative value per floristic plot per site (Table S3).

### 2.4 Determination of Lemur Species Presence

We conducted three diurnal and three nocturnal systematic surveys along each 1.2 km transect to assess lemur presence across its different sections (Peres 1999). Four observers walked each transect quietly at approximately 0.5 km/h (Rakotondravony and Radespiel 2007), continuously scanning both sides for animals. Surveys were conducted on three consecutive days in the morning (06.30–08.30) for diurnal species and in the evening (18.15–20.30) for nocturnal species, using headlamps to detect eye‐shine. Cathemeral species were recorded whenever encountered. We applied a maximum detection width of 50 m on either side of the transect line and recorded all lemurs detected within this zone, with encounters being mapped to the nearest 10 m along the transect line. While diurnal species were directly detected visually, all nocturnal species were reliably detected first indirectly but also visually through their eye‐shine reflection generated by the tapetum lucidum (Wolin and Massopust 1970) under headlamp illumination, and then taxonomically identified by means of a stronger torch. Because datasets for each species were analyzed independently, interspecific differences in detectability did not confound our statistical comparisons. Importantly, however, perpendicular detection distances did not differ significantly between burnt and unburnt zones (Rabemananjara et al. 2025), indicating that burn status did not systematically bias the detectability of species during surveys.

### 2.5 Statistical Analysis

Because of very small numbers of observations, statistical models could not be fitted for *P. coquereli* and *E. mongoz* (Rabemananjara et al. 2025). *C. medius* undergoes hibernation during much of the dry season (May–September) (Müller and Thalmann 2002) and was consequently included in the analysis only for 11 sites that were surveyed from 5 September onwards in both years. However, the number of encounters was still rather small for the six remaining lemur species due to the limited number of surveys per site and the limited length of the transect partitions with different fire history per site, and furthermore showed little variation across floristic plots. Therefore, we used the raw encounter data to derive lemur presence records as a binary proxy for confirmed habitat use in our statistical models. This standardized binary response variable allowed robust comparisons of species‐specific occurrence probabilities across transect partitions characterized by different fire histories for six lemur species. All species‐specific models and those for lemur species richness and fire‐vegetation interaction were fitted in R (R Core Team 2025) via the RStudio interface (Posit Team 2025).

### 2.6 Modeling the Effects of Vegetation Structure and Abiotic Factors on Lemur Presence and Lemur Species Richness

Before fitting models, we tested for correlation among the structural variables using Pearson's correlation (“stats” package). Since height class counts and DBH class counts were strongly correlated (*r* ≥ 0.7, Figure S2), we chose the height class counts to represent both in the principal component analysis (PCA). We used the PCA to reduce multicollinearity and to condense the eight remaining parameters included in the vegetation data set (four height classes, woody plant density, three forest stratum cover estimates). The PCA involved standardizing data, calculating the covariance matrix, and extracting Eigenvalues > 1 to identify principal components (PCs) used for downstream analyses (Table S4). Vegetation variables with loadings > 0.3 were considered important (Table S4) (Hair et al. 2019). Prior to fitting the final models, Pearson's correlation coefficients were also calculated between the retained PCs, woody species richness, and abiotic variables (maximum temperature and relative humidity) to check for multicollinearity among all predictors included in the global model (Figure S3). We fitted Generalized Linear Mixed Models (GLMMs) with the “glmmTMB” package (Brooks et al. 2017) to test how vegetation structure (main PCs), abiotic factors (humidity, maximum temperature), and woody species richness (species per plot) impacted the presence/absence of each lemur species and lemur species richness (response variables), respectively. Based on the data structure and distribution of the response variable, we applied binomial model families. Model fit was evaluated with the “DHARMa” package (Hartig and Hartig 2022), which included testing for residual distribution, over‐/underdispersion, outliers, and zero inflation. If required, we included ziformula() and dispformula() to address zero inflation and dispersion issues, respectively (Brooks et al. 2017). The inclusion of both month and site and of site alone as a random effect caused convergence problems; therefore, site was excluded as a random factor from the final model.

We used an information‐theoretic approach for model selection with the dredge() function in the “MuMIn” package (Bartoń 2022). This generates all possible subsets of the global model and ranks them by AICc. Models within ΔAICc ≤ 2 of the best model were considered equally competitive and well supported (Burnham and Anderson 2002), and were retained for further analysis (Burnham and Anderson 2002; Table S5). To account for model uncertainty, we applied conditional model averaging with the model.avg() function, restricted to the well‐supported set of models with ΔAICc ≤ 2 (Burnham and Anderson 2002; Dormann et al. 2018; Le and Clarke 2022). This method averages estimates only across models where a variable appears, reducing dilution from absent variables (Symonds and Moussalli 2011), and is preferred over selecting a single best model because it incorporates uncertainty from multiple plausible models. Model‐averaged coefficients, standard errors, and 95% confidence intervals (CIs) were calculated, and each variable's importance (RI) was assessed from the summed Akaike weights across the top model set (ΔAICc ≤ 2) in which the variable was contained. Variables with importance values ≥ 0.8 are considered strongly supported, while those ≥ 0.5 are interpreted as having moderate support, but were considered only if the respective CIs did not contain zero (Burnham et al. 2011; Symonds and Moussalli 2011).

In view of the used model‐averaging approach, we decided to report effect sizes and their 95% CIs for all analyses instead of *p*‐values. Thereby, the interpretation will be based on the magnitude and precision of the biological effects rather than on their significance level (Symonds and Moussalli 2011).

### 2.7 Modeling the Effects of Fire History on Relevant Vegetation Structure and Abiotic Conditions

For our second objective, we examined the influence of the fire‐related variables (fire frequency, time since the last fire, maximum fire severity, burn status [burnt vs. unburnt]) on those relevant vegetation structures that were identified in our first objective and thereby relevant to lemurs. We fitted two GLMMs using two datasets for each dependent variable with the “glmmTMB” package. The first dataset (Model A) included all transect partitions across all sites and was used to examine the effect of the burn status (burnt/unburnt) and of the number of fires (0–6) on the vegetation parameters and abiotic conditions. The second dataset (Model B) included only all burnt partitions and was used to evaluate the impact of the number of years since the last fire and the maximum fire severity on vegetation parameters. Preliminary analyses revealed no significant difference between burnt and transition zones regarding the vegetation and abiotic parameters (Table S11). Therefore, we combined the transition and burnt transect zones into a single “burnt” category. As our response variables were normally distributed, we selected the Gaussian family for modeling. Both site and month were included as random effects. Results are reported using *p*‐values, with statistical significance determined at *p* < 0.05.

## 3 Results

We retained three PCs with Eigenvalues ≥ 1 in the PCA that together explained 68.45% of the total variance (Table S4). PC1 (29.67% explained variance, Figure S4), interpreted as overall forest structural complexity, reflects a gradient from structurally simple to complex forest stands, with strong positive loadings for woody plant density (+0.354), counts of medium‐height trees (+0.307), tall trees (+0.461), and very tall trees (+0.405), as well as high cover of the medium stratum (+0.463) and upper stratum (+0.373). Plots with high positive PC1 scores are therefore characterized by dense, multi‐layered canopies dominated by tall trees, while plots with low PC1 scores represent more open, less structurally complex forests. PC2 (23.87% explained variance, Figure S6), interpreted as understory and mid‐story openness, describes the density of the two lower vegetation strata, with strong negative loadings for counts of small trees (−0.539) and medium trees (−0.502), and lower stratum cover (−0.485). Plots with high negative PC2 scores are characterized by dense understory and mid‐story vegetation, whereas positive PC2 scores indicate more open lower strata with greater upper canopy dominance. Finally, PC3 (14.9% explained variance, Figure S5), interpreted as vertical forest stratification, captures a contrast in forest layering, where high positive scores reflect a well‐developed two‐layered structure, characterized by high counts of very tall trees (+0.510), larger upper stratum cover (+0.477), and a developed understory (+0.467), but a less prominent medium stratum (−0.400). Negative PC3 scores, by contrast, reflect plots with a more homogeneous mid‐canopy structure dominated by medium tree counts and cover, with limited vertical differentiation. These three PCs were subsequently used as structural predictors in the species‐specific GLMMs.

### 3.1 Influence of Vegetation Structure and Abiotic Factors on the Presence of Large‐Sized Lemurs

**Figure 2:**
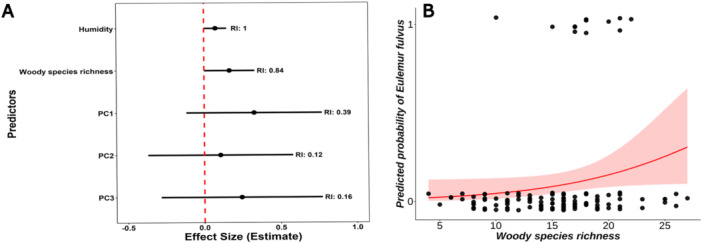
(A) Averaged parameter estimates for predictors of *Eulemur fulvus* presence that were included in the subset of best models. Points represent effect size estimates on the logit scale, with horizontal whiskers showing 95% confidence intervals. Important variables are those that do not cross the dashed line with their confidence intervals and have an RI ≥ 0.8. Relative Importance (RI) values, based on the sum of Akaike weights across best models, are displayed for each predictor. (B) Relationship between woody species richness and the predicted probability of *E. fulvus* occurrence. Individual data points are displayed as jitter, and the fitted probability line (± 95% CI) illustrates a positive association between increasing woody species richness and the likelihood of *E. fulvus* presence within surveyed habitats.

The analysis identified humidity and woody species richness as influential predictors for the presence of *E. fulvus* (Estimate_humidity_ = 0.07 ± 0.03 SE, 95% CI = [0.002, 0.148], RI = 1.00; Estimate_woody_sp_richness_ = 0.16 ± 0.08 SE, 95% CI = [0.0005, 0.3308], RI = 0.84, Figure 2A). In other words, the probability of *E. fulvus* presence increased with higher humidity levels and higher woody species richness (Figure 2B). In contrast, the vegetation structure PCs (PC1–PC3) had low importance, and CIs overlapped zero (Figure 2, Table S6), while maximum temperature was not even included in the top models (Table S6).

### 3.2 Influence of Vegetation Structure and Abiotic Conditions on the Presence of Medium‐Sized Lemurs

**Figure 3:**
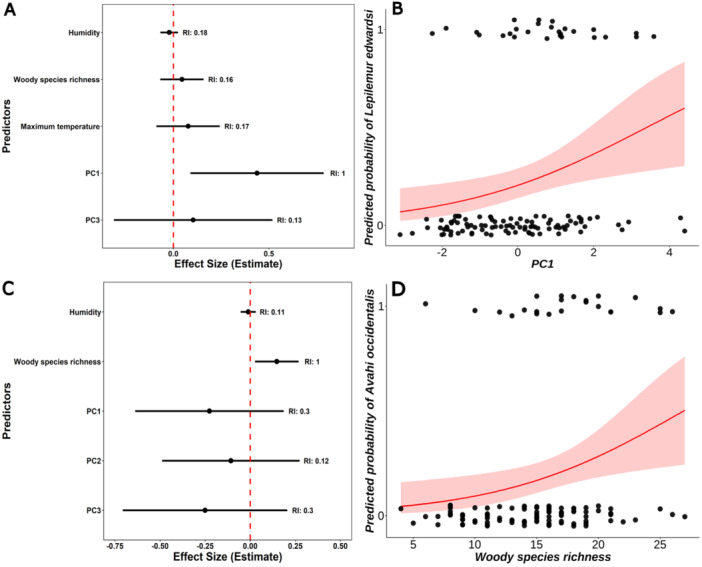
Averaged parameter estimates for predictors for the presence of the two medium‐sized lemurs, *Lepilemur edwardsi* (A) and *Avahi occidentalis* (C). Effect sizes are displayed on the logit scale for each parameter that was included in the averaged best model, with horizontal whiskers representing 95% confidence intervals. Important variables are those whose confidence intervals do not cross the dashed line and have an RI ≥ 0.8. Relative Importance (RI) values, based on the sum of Akaike weights across best models, are displayed for each predictor. (B) Relationship between PC1 and the predicted probability of *L. edwardsi* occurrence. Individual data points are displayed as jitter, and the fitted probability line (± 95% CI) illustrates a positive association between increasing structural complexity and tall forests and *L. edwardsi* presence. (D) Relationship between woody species richness and the predicted probability of *A. occidentalis* occurrence. Individual data points are displayed as jitter, and the fitted probability line (± 95% CI) illustrates a positive association between increasing woody species richness and the likelihood of *A. occidentalis* presence.

Forest structural complexity (PC1) was the only important predictor for the presence of *L. edwardsi*, showing a positive relationship with species presence (Estimate_PC1_ = 0.44 ± 0.18 SE, 95% CI = [0.09, 0.79], RI = 1.00, Figure 3A). Since PC1 signaled overstory complexity and closure, *L. edwardsi* presence was associated with structurally complex and tall forests (Figure 3B). The other variables (humidity, maximum temperature, woody species richness, PC3) had low importance, and CIs overlapped zero, or they were not even included in the best model set (PC2, Table S7).

Woody species richness was the only important predictor for the presence of *A. occidentalis*, positively affecting its occurrence probability (Estimate_woody_sp_richness_ = 0.15 ± 0.06 SE, 95% CI = [0.03, 0.27], RI = 1.00, Figures 3C,D). Other predictors (PC1, PC3, humidity) showed low relative importance, while maximum temperature was not even included in the best model set (Table S7).

### 3.3 Influence of Vegetation Structure and Abiotic Conditions on the Presence of Small Lemurs

**Figure 4:**
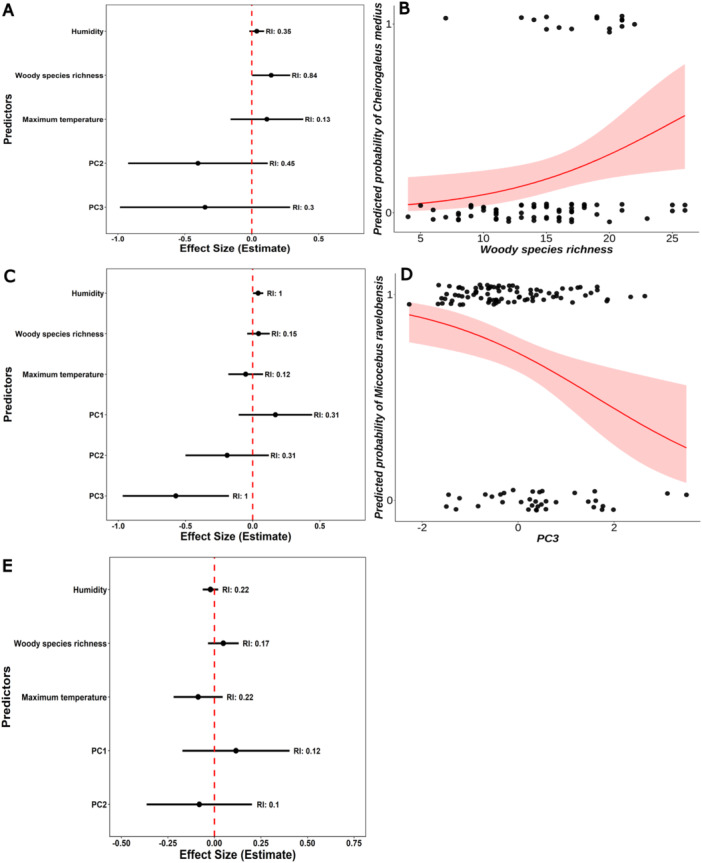
Averaged parameter estimates for predictors for the presence of the small‐sized lemurs *Cheirogaleus medius* (A), *Microcebus ravelobensis* (C), and *Microcebus murinus* (E). Effect sizes are displayed on the logit scale for each parameter that was included in the averaged best model, with horizontal whiskers representing 95% confidence intervals. Important variables are those whose confidence intervals do not cross the dashed line and have an RI ≥ 0.8. Relative Importance (RI) values, based on the sum of Akaike weights across best models, are displayed for each predictor. (B) Relationship between woody species richness and the predicted probability of *C. medius* occurrence. Individual data points are displayed as jitter, and the fitted probability line (± 95% CI) illustrates a positive association between increasing woody species richness and *C. medius* presence. (D) Relationship between PC3 and the predicted probability of *M. ravelobensis* occurrence. Individual data points are displayed as jitter, and the fitted probability line (± 95% CI) illustrates a positive relationship between dense middle stratum forest (negative PC3 values) and the likelihood of *M. ravelobensis* presence.

Woody species richness had an important positive effect on the probability of occurrence of *C. medius* along the transects (Estimate_woody_sp_richness_ = 0.14 ± 0.07 SE, 95% CI = [0.002, 0.287], RI = 0.83, Figures 4A,B). Other predictors, such as PC2, PC3, humidity, and maximum temperature, were retained in the averaged best model but were not important predictors, while PC1 was not even included in the best averaged model (Table S8). Humidity and PC3 emerged as the strongest predictors of presence for *M. ravelobensis* (Estimate_humidity_ = 0.04 ± 0.01 SE, 95% CI = [0.002, 0.079], RI = 1.00; Estimate_PC3_ = −0.57 ± 0.19 SE, 95% CI = [−0.966, −0.176], RI = 1.00, Figure 4C). Higher occurrence probabilities for this species were therefore associated with more humid forests and with habitats characterized by a dense middle stratum (Figure 4D). The other predictors, namely PC1, PC2, woody species richness, and maximum temperature, were not influential (Table S8). In the case of *M. murinus*, no vegetation or abiotic predictor impacted its occurrence probability. All parameters besides PC3 were included in the best averaged model, but their effects were only weak (Figure 4E, Table S8).

### 3.4 Influence of Vegetation Structure and Abiotic Conditions on Lemur Species Richness

**Figure 5:**
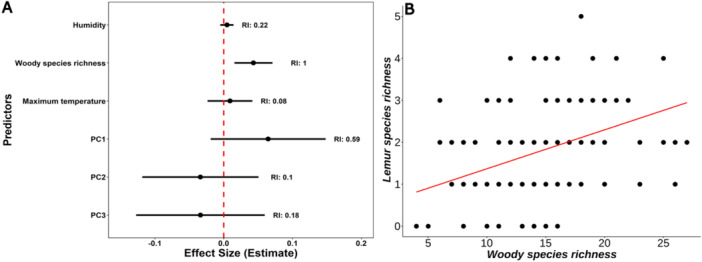
(A) Model‐averaged parameter estimates for predictors of lemur species richness. Points represent effect size estimates on the logit scale, with horizontal whiskers indicating 95% confidence intervals. Relative Importance (RI) values, calculated from the sum of Akaike weights across relevant candidate models, are displayed next to each predictor. (B) Relationship between woody species richness and lemur species richness. Individual data are displayed (points), and a regression line (± 95% CI) illustrates a positive association between increasing woody species richness and lemur species richness.

Woody species richness emerged as the most important predictor of lemur species richness and had a positive and significant effect (Estimate _woody_sp_richness_ = 0.04 ± 0.01 SE, 95% CI = [0.015, 0.070], RI = 1.00, Figures 5A,B). The other predictors, that is, PC1, PC2, PC3, humidity, and maximum temperature, exhibited lower relative importance and CIs overlapping zero, indicating limited explanatory power for lemur species richness (Table S9).

### 3.5 Impact of Fire History on Relevant Vegetation Parameters and Abiotic Factors

#### 3.5.1 Forest Humidity and Woody Species Richness

Forest humidity was not strongly influenced by the fire history across all sites. None of the variables showed a detectable association with humidity (Table S9). These results indicate that forest humidity did not differ systematically between burnt and unburnt plots and did not clearly respond either to fire frequency, the time since last fire, or to the maximum fire severity.

In contrast, woody species richness was affected by the fire history. Specifically, it was higher in unburnt than in burnt plots (Estimate_unburnt_ = 5.05 ± 1.16 SE, 95% CI = [2.77, 7.32], *p* < 0.0001, Figure S7A), and maximum fire severity had a strong negative effect on woody species richness (Estimate_max_severity_ = −33.1 ± 8.25, 95% CI = [−49.2, −16.9], *p* < 0.0001, Figure S7B). In contrast, neither the number of fires nor the time since the last fire showed a detectable effect on woody species richness (Table S10).

#### 3.5.2 Complexity, Density, and Cover of the Vegetation (PC1, PC3)

We modeled two structural vegetation parameters (PC1, PC3) to assess fire effects on those forest architectural parameters that were shown to be relevant to lemurs in the previous analytical steps. PC1 captured overstory complexity and closure and was affected by three fire variables. Unburnt sites had higher PC1 scores (Estimate_unburnt_ = 1.11 ± 0.33 SE, 95% CI = [0.46, 1.75], *p* < 0.001, Figure S8A), that is, a higher overstory complexity and closure than burnt sites (Table S11). Moreover, time since the last fire was positively related to PC1 (Estimate_last fire_ = 0.05 ± 0.01 SE, 95% CI = [0.0246, 0.0775], *p* = 0.0001, Figure S8B), while maximum fire severity had a strong negative effect (Estimate_max_severity_ = −10.5 ± 0.62 SE, 95% CI = [−15.6, −5.39], *p* < 0.0001, Figure S8C) showing that severe fires greatly reduced canopy complexity.

Neither burnt status (unburnt/burnt), fire frequency, nor maximum fire severity showed an impact on PC3 (Table S10). Only the time since the last fire showed a small positive influence on PC3, that is, the density and cover of very tall and small trees (Estimate_last fire_ = 0.02 ± 0.01 SE, 95% CI = [0.002, 0.047], *p* = 0.03, Figure S9).

## 4 Discussion

### 4.1 Impact of Fire‐Modulated Vegetation Structure and Abiotic Factors on the Large *E. fulvus*

Across ANP, *E. fulvus* exhibited a clear preference for humid forests and high woody species richness. While relative humidity remained consistent across sites with different fire histories, woody species richness peaked in unburnt areas and declined with increasing fire severity. These patterns align with those reported by Rabemananjara et al. (2025), who found *E. fulvus* mainly in unburnt zones or in forests with more than two decades of post‐fire recovery. Under such conditions, humid valleys may function as partial fire refugia, where riparian vegetation and higher soil moisture may act as natural firebreaks and help preserve essential resources.

As a mainly frugivorous lemur species (Mittermeier et al. 2023), the preference for forests with high woody species richness suggests that *E. fulvus* presence may be constrained by the presence of a diverse spectrum of food species that may provide some fruits even during the lean dry season (Sato 2013). Intense fires appear to reduce the woody species richness, suggesting that declines in key food resources may limit the species' ability to persist in burned landscapes. *E. fulvus* may be forced to respond with “habitat shifting,” a strategy that was already observed in response to temporary food scarcity (Sato 2013). During “habitat shifting,” a social group temporarily leaves the established home range to exploit distant habitats with higher food availability (Sato 2013). Such a strategy is certainly facilitated by the lack of territoriality in *E. fulvus* (Johnson 2007), although it may also lead to permanent home range shifts and potential crowding phenomena in view of the rather slow post‐fire habitat dynamics (Rabemananjara et al. 2025).

Beyond direct food availability, *E. fulvus* is a strictly arboreal and cathemeral species that spends the majority of its time in the canopy or lower levels of trees, with only around 2% of time spent on the ground (Tanaka 2007), likely making the availability and connectivity of canopy structures a critical requirement for daily movement and predator avoidance. *E. fulvus* lives in social groups ranging from a few individuals to over a dozen (Mittermeier et al. 2023), and the maintenance of cohesive social groups requires sufficiently large and structurally complex forest patches to accommodate group home ranges without excessive intra‐specific feeding competition inside social groups or with neighboring groups. In disturbed or degraded habitats, reduced canopy connectivity and simplified forest structure may therefore also impact group cohesion and inter‐group spacing.

Finally, *E. fulvus* plays a critical ecological role as the sole disperser of large‐seeded tree species (> 10 mm diameter) in northwestern Madagascar, dispersing seeds of up to 70 plant species (Sato 2012), making it a keystone mutualist whose presence is tied to the maintenance of floristic diversity. This generates a reciprocal relationship *between E. fulvus* and woody species richness: floristically diverse forests provide the variety of fruiting species that sustain *E. fulvus* throughout seasonal cycles, while *E. fulvus* in turn supports the regeneration and maintenance of various tree species, including those with large seeds, through seed dispersal. Fire‐driven reduction in woody species richness, therefore, likely not only diminishes the food availability for *E. fulvus* but may also compromise the long‐term regeneration potential of large‐seeded trees, creating a self‐reinforcing negative cycle of floristic impoverishment in heavily burnt landscapes, highlighting the need for future studies quantifying food availability, seed dispersal ecology, and fire effects in these vulnerable dry forests.

### 4.2 Impact of Fire‐Modulated Vegetation Structure and Abiotic Factors on Medium‐Sized Lemurs

The presence of *L. edwardsi* in the dry deciduous forest of ANP strongly depended on the availability of complex vertical forest structures and mature canopy elements (i.e., higher values of PC1), but not on woody species richness. With the exception of three sites that had experienced rather recent fires (1–5 years earlier), this species was restricted to unburnt areas or to older fire zones (Rabemananjara et al. 2025) where the upper strata had already regenerated (Rasolozaka et al. in revision). These findings are consistent with the species' reliance on suitable substrates for its characteristic clinging‐and‐leaping locomotion (Warren 1997) and on secure shelters provided by large overstory trees (Rasoloharijaona et al. 2003; Warren 1997). This species depends on tall, mature trees with large vertical trunks to execute its rapid and energy‐efficient leaping between trunks and branches (Warren 1997), and the loss of such structurally complex trees in burnt areas may severely restrict movement options, forcing individuals to make longer, more exposed leaps or to descend to lower and more exposed strata to navigate the landscape. The replacement of tall mature trees by shorter and more sparsely distributed regenerating vegetation in burnt forests further reduces canopy cover and vegetation density (Smit et al. 2010), substantially increasing the visibility of individuals and reducing their crypticity, making them more detectable to their main predators that include the fossa (*Cryptoprocta ferox*), the Madagascar harrier‐hawk (*Polyboroides radiatus*), and the Malagasy boa (*Acrantophis madagascariensis*) (Fichtel 2007). The combined effect of compromised locomotor substrates, restricted movement options, lack of suitable tree holes for resting, reduced crypticity, and increased predation exposure, alongside the greater energetic costs associated with movement through structurally simplified habitats (Warren and Crompton 1997), may therefore make burnt forests particularly inhospitable for this species.

Taken together and in contrast to *E. fulvus*, our results suggest that access to appropriate substrates for locomotion and resting is likely of greater ecological relevance for this nocturnal folivore than floristic parameters. Future studies should investigate how fire history shapes dietary characteristics, substrate use, and shelter ecology in *L. edwardsi*.

The occurrence of *A. occidentalis* was positively related to a higher woody species richness, demonstrating the role of floristic diversity for this more specialized folivore (Thalmann 2001). Fires and fire intensity reduced woody species richness, and fires also reduced the complex vertical canopy structure (PC1), which may both have synergistically limited nutritional options for *A. occidentalis*, which depends on diverse diets to cope with toxic plant secondary compounds (Thalmann 2001). Fire‐driven floristic simplification often coincides with an enrichment of fewer fire‐tolerant tree species and a reduction in abundance of preferred food plants (Bargali et al. 2022). Our study showed that the fire‐related floristic simplification in the ANP does severely impact *A. occidentalis* and cannot be compensated by flexible dietary shifts of this species. The response of *A. occidentalis* to changes in woody species richness resembles that of *E. fulvus*. However, it is not yet clear which of the fire‐sensitive tree species are keystone resources and drive the strong floristic response of these two lemur species, and this question should be addressed in future work. Rabemananjara et al. (2025) showed that *A. occidentalis* and also *E. fulvus* are more regularly observed again after > 23 years post‐fire, suggesting that woody species richness can recover if no additional fire impacts the forest in the meantime. Such slow post‐fire regeneration has also been observed in other woody assemblages (Allerton et al. 2025).

It is important to emphasize that our data represents a snapshot in time and does not allow conclusions about population trends or long‐term demographic consequences of fire for any of the species. Overall, the results for *L. edwardsi* and *A. occidentalis* support hypotheses H1 and H2 in a nuanced way. H1 predicted that lemur presence increases with greater structural complexity, canopy connectivity, and floristic diversity, while H2 predicted that these parameters are compromised by fires. While the presence of one medium‐sized folivore (*L. edwardsi*) relied mainly on mature, multilayered forests with a well‐covered canopy, underscoring the importance of intact vertical structure for their persistence in fire‐modulated landscapes, the presence of the other folivore (*A. occidentalis*) depended mainly on a high woody species richness, that is, on floristic diversity. This diversity was considerably impacted by past fires, likely constraining the dietary spectrum of available food plants. Neither species was strongly impacted by humidity or temperature variations in the forest. This negligible role of abiotic factors may be best explained by the long‐term territoriality of both species that is maintained despite substantial seasonal variations in abiotic conditions (Ramanankirahina et al. 2012; Rasoloharijaona et al. 2003). It is therefore suggested that changes in temperature or humidity rather led to changes in microhabitat use, resource use, or other adaptive behavioral changes in these two species, but not directly to changes in territory occupancy and, therefore, presence or absence at a given site.

### 4.3 Impact of Fire‐Modulated Vegetation Structure and Abiotic Factors on Small Lemurs

The probability of occurrence of *C. medius* strongly depended on woody species richness and increased with increasing floristic diversity, which in turn was shown to decline in response to fires and increasing fire severity. Conversely, the other ecological parameters did not show important effects on the presence of *C. medius*. This result closely resembles the findings for *A. occidentalis*, despite their contrasting diet, which is largely omnivorous in the case of *C. medius* (Fietz and Ganzhorn 1999). However, floristically diverse forests likely provide reliable, though seasonally variable, food resources (e.g., fruits, flowers, seeds, gum, leaves) even during the lean season of the year (Garcia et al. 2014). The availability of a broad spectrum of woody species and food items may be particularly relevant for this lemur species, as it is an obligate hibernator that spends more than 5 months of the dry season inactive before the beginning of the mating season (Fietz and Ganzhorn 1999; Lahann 2007; Souza‐Alves et al. 2021). Due to a massive loss in body mass during hibernation (Dausmann et al. 2004), it can be expected that energy requirements will be high after arousal, and that immediate and reliable access to energy‐rich food resources may have important fitness consequences (Blanco et al. 2021). Under these conditions, choosing territories that contain a high variety of woody species may provide a reliable “resource insurance”, allowing even to buffer year‐to‐year uncertainties in food availability. Our previous work did not find strong impacts of fire variables on the abundance of *C. medius*, although a statistical trend was detected for increasing abundance with increasing fire age (Rabemananjara et al. 2025). However, floristic diversity was not quantified in that study, and the feeding ecology of *C. medius* in this forest region is still poorly documented. Therefore, further research is needed to clarify whether the presence of *C. medius* in forest sites is primarily driven by the distribution of certain keystone food resources or potentially also by the availability of certain cavity‐bearing tree species that facilitate well‐insulated and protected nesting, torpor, and hibernation of small family groups that typically rest and hibernate together during 5 months of the year (Lahann 2007; Müller 1998). Although *C. medius* was observed also in a few burnt and degraded habitats (Hending 2021; Rabemananjara et al. 2025; Steffens et al. 2020), its presence in these disturbed habitats may represent transient use rather than long‐term persistence and may go along with reduced population densities and lower reproductive rates in unfavorable environments (Fietz et al. 2000; Lehman et al. 2006; Schäffler et al. 2015).

The presence of *M. ravelobensis* reflected an affinity to humid microclimates and the importance of a well‐covered middle stratum (5–10 m) for this species (negative effect of PC3). The importance of mesic conditions for this species was already pointed out in earlier work across the ANP (Rakotondravony and Radespiel 2009) and largely corresponds to topographically driven microhabitat preferences of this species (Rabemananjara et al. 2025; Steffens et al. 2022). An affinity for a well‐developed middle stratum may relate to different aspects of its biology. First, a well‐covered middle stratum may contain a large number of suitable sleeping sites for *M. ravelobensis*, as it is known that they use a wide variety of shelter types, among which also dense tangles of lianas and even self‐built leaf nests that can be found at these heights, even in unburnt forest (Radespiel et al. 2003, Thorén et al. 2010). Second, a well‐covered middle stratum may provide valuable protection from predators for small species like mouse lemurs (Torre et al. 2023). Our study also showed that this structural parameter (PC3) was influenced by the time since the last fire. Specifically, fires led to a higher‐cover middle stratum in more recently burned forests, which was reduced in favor of the small and large strata with increasing age of the fire. Such a well‐covered middle stratum in recent fire zones may explain why the two smallest lemurs were equally likely found in all sites, whether they were burnt or unburnt (Rabemananjara et al. 2025). This resilience towards fires is consistent with the adaptive habitat flexibility reported in small‐bodied lemurs inhabiting disturbed habitats (Hending 2021). To what extent this habitat flexibility is the result of flexible resource use and dietary shifts in these omnivorous solitary foragers has to be clarified in future studies.

*M. murinus* also exhibited a strong fire‐related ecological resilience, as its probability of occurrence did not vary systematically in response to changes in vegetation structure, woody species diversity, or abiotic conditions. Its persistence across fire‐modulated habitats reflects generalist foraging habits and a flexible use of fine‐branch microhabitats that persist even in regenerating forests (Hending 2021). Structural changes and temporary openings in the vegetation may not challenge this species, which readily exploits secondary growth and even small forest fragments for shelter and feeding (Andriatsitohaina et al. 2020; Steffens et al. 2022). Its wide dietary breadth that includes arthropods, insect secretions, fruits, and gum, further enhances survival in modified post‐fire environments (Ganzhorn 2003; Ganzhorn and Schmid 1998; Radespiel et al. 2006).

The results on both mouse lemur species align with hypothesis H2, which predicted that the influence of fire‐related vegetation changes on lemur presence varies with body size; that is, small, generalist lemurs are more tolerant of fire‐modulated changes than larger taxa. As a consequence, small‐bodied lemur species may function as ecological bridge species, sustaining key processes such as seed dispersal (Ramananjato 2025), pollination, and invertebrate predation during early post‐fire recovery phases when larger, canopy‐dependent species are absent (Ganzhorn et al. 2024). Their persistence across varying fire histories underscores the adaptive capacity and ecological resilience of small‐bodied generalists.

### 4.4 Lemur Species Richness: The Influence of Fire‐Modulated Floristic Diversity in Structuring Lemur Assemblages

Because several species were encountered only rarely, a complete assessment of the lemur assemblage was not feasible, thereby constraining our capacity to evaluate fire effects at the community level. The scarcity of observations for the two rarest species (*P. coquereli* and *E. mongoz*) leaves substantial gaps in our understanding of how fire regimes influence their populations and occurrence. *P. coquereli* was observed predominantly in unburnt forests or in areas with fires older than two decades, while *E. mongoz* exhibited a similarly restricted distribution, occurring along transects at only one of 18 sites and almost exclusively in unburnt habitat (Rabemananjara et al. 2025). Evaluating their fire responses in more detail will require intensive and sustained monitoring efforts designed to improve detection probabilities for rare species.

When interpreting these patterns, it is important to consider the historical role of fire in shaping this ecosystem. Although Malagasy forests were not originally fire‐prone, human arrival on the island has substantially increased fire frequency, likely driving significant changes in vegetation structure and composition since around 1900 AD (Razanatsoa et al. 2022). Recurrent fire exposure may have progressively favored a few fire‐tolerant tree species and grasses while reducing the abundance and diversity of fire‐sensitive tree species (Bargali et al. 2022). Importantly, previous work conducted in our study area suggests that forests subjected to frequent fires effectively stop regenerating, a process that may be largely irreversible (Percival et al. 2024). Such long‐term fire‐driven floristic shifts may have cascading consequences for the lemur community, as the gradual replacement of diverse, resource‐rich forest by structurally and floristically simplified vegetation could reduce the carrying capacity of the landscape for more specialized lemur species. The finding that fire severity rather than fire frequency or interval appeared to be the primary driver of woody species richness loss further suggests that even a single intense fire event may be sufficient to cause lasting floristic impoverishment, independent of fire history per se.

The results for the better‐sampled species suggest that fire history shapes lemur habitat use and distribution in the dry deciduous forests of northwestern Madagascar. Woody species richness emerged as the strongest predictor of overall lemur species richness, which may underscore the central role of species‐rich plant communities in maintaining multi‐species primate assemblages (Ganzhorn 1999; Schüßler et al. 2018). This relationship appears to be driven by similar patterns observed in three lemur species (*E. fulvus, A. occidentalis, C. medius*), as higher woody species richness may provide a wider array of fruiting, flowering, and leaf phenologies that could sustain species with dietary constraints and microhabitat preferences throughout challenging seasonal cycles of variable food availability (Ganzhorn et al. 1997). Floristically diverse forests may also lead to greater structural complexity, potentially providing a wider variety of canopy strata, substrates, and microhabitats that could accommodate the different locomotor, dietary, and sheltering requirements of a diverse lemur community (Ganzhorn 1999). Woody species richness may therefore be important not only as a direct mediator of stable food availability, but also as an indirect proxy for structural habitat continuity and complexity. In the dry deciduous forests of northwestern Madagascar, fruiting tree diversity may be particularly critical, as the diversity of fruiting phenologies across the annual cycle could provide a more continuous and reliable food supply for frugivorous and omnivorous lemurs during periods of resource scarcity (Sato 2013; Ganzhorn et al. 1997). Fire‐tolerant woody species may still provide some food resources in recently burnt areas, potentially explaining the flexible use of burnt habitats by dietary generalist species such as *C. medius* and mouse lemurs. However, these fire‐tolerant woody species are unable to fully compensate for the loss of fire‐sensitive, resource‐rich tree species that characterize intact forests, and their nutritional value and palatability for lemurs remain to be investigated in future studies.

The distribution of lemur species across fire‐modified landscapes may also be shaped by interspecific competition and niche separation among sympatric species. In intact forests, co‐occurring lemur species are thought to partition resources and space along multiple ecological axes, including activity, diet, body size, locomotion style, and vertical canopy stratum use, thereby reducing direct competition (Ganzhorn 1999). Fire‐driven simplification of forest structure and floristic diversity likely impoverishes these niche dimensions, potentially intensifying competitive interactions and likely excluding more specialized locomotion types from degraded habitats. The strong dependence of *L. edwardsi* on intact upper canopy structures contrasts markedly with the more flexible microhabitat use of mouse lemurs, which appear capable of exploiting a wider range of vertical strata, including the middle stratum that develops rapidly in recently burnt forests.

The observation that only two of eight lemur species showed considerable resilience to fire‐related vegetation disturbance underlines the conclusion that the dry deciduous forests of northwestern Madagascar and its lemur inhabitants are not well adapted to fire‐related vegetation changes and have likely not evolved under this selection pressure of recurrent fires over evolutionary timescales. Taken together, these findings suggest that conserving floristically diverse and structurally complex forests may be essential for maintaining full lemur assemblages across fire‐prone landscapes, and that woody species richness could serve as an integrative proxy for lemur habitat quality (Huang et al. 2018) across the fire‐modified dry deciduous forests of northwestern Madagascar.

### 4.5 Study Limitations

Several limitations of this study should be acknowledged when interpreting the results. The occurrence data collected across the 18 study sites represent a cross‐sectional snapshot and therefore did not capture the full spatial and temporal dynamics of lemur movement and space use across the landscape. No population dynamics, such as demographic trends, reproductive rates, or survival rates, were quantified, and consequently, the observed associations between fire history, vegetation structure, and lemur occurrence should be interpreted as habitat use at the time of sampling rather than as direct evidence of overall population‐level processes. Additionally, two lemur species (*P. coquereli* and *E. mongoz*) were encountered too rarely to be included in the species‐specific modeling, and the real number of fire‐sensitive species may therefore be higher than suggested by our models. Despite these limitations, this study provides a valuable overview of the impacts of fire on a diverse lemur community inhabiting a fire‐prone dry deciduous forest. By combining species‐specific modeling with detailed vegetation structural data and woody species richness, our results offer important insights into how fire‐modulated changes in forest structure and floristic diversity shape lemur habitat use and community composition, providing a useful baseline for future long‐term monitoring and conservation planning in ANP and similar fire‐prone forest ecosystems across Madagascar.

## 5 Conservation Implications

Our findings from ANP underscore that fire is not only a driver of vegetation change but also a critical determinant of lemur community composition. By altering tree diversity, canopy complexity, and floristic composition, recurrent and severe fires progressively erode the ecological foundations that sustain lemur populations. Although only one large‐bodied lemur species was represented in our study, its consistent restriction to humid, species‐rich forests underscores its vulnerability to fire‐driven habitat degradation. In addition, medium‐bodied taxa declined in repeatedly burnt areas, while smaller dietary and locomotor generalists persisted across a wider range of fire histories. Importantly, lemur species richness depended on higher woody species diversity, emphasizing that areas already affected by multiple fires that are impoverished in woody species diversity and now widespread across ANP, support fewer lemur species. As large sections of the protected area have burned in recent decades (Rasolozaka et al. 2025), these spatial trends highlight an urgent conservation concern: fire is progressively concentrating lemur diversity into a shrinking set of less‐disturbed forest patches, making the maintenance and protection of these remaining habitats and lemur species increasingly critical.

Given the centrality of vegetation structure and floristic diversity for lemur community organization and the prominent threats imposed by fires to the integrity of the dry deciduous forests of western Madagascar and of ANP (Frappier‐Brinton and Lehman 2022; Rasolozaka et al. 2025), effective fire management is urgently needed for all protected zones in Madagascar's dry forest ecosystems. Priority actions should include: (1) long‐term monitoring of vegetation recovery and lemur assemblage dynamics across fire‐history gradients, which is essential for tracking population trends of fire‐sensitive species such as *P. coquereli, E. mongoz*, and *L. edwardsi*, and for evaluating whether lemur communities can recover as forests regenerate; (2) protection of mesic forested valley systems that may serve as fire refugia for humidity‐dependent species such as *E. fulvus* and *M. ravelobensis*, preserving the source populations needed for recolonization of recovering burnt areas; (3) maintenance of structurally complex, mature canopy forest in old‐growth and long‐unburnt areas, which is critical for locomotor and dietary specialists such as *L. edwardsi* that depend on large trees for movement, shelter, and predator avoidance, and for *A. occidentalis* or *C. medius* that depend on resource‐rich floristically diverse tree communities; (4) prevention of fire recurrence in recovering burnt areas, as repeated fires severely hinder forest regeneration and have been shown to reduce both woody species richness and lemur species richness below recovery thresholds (Percival et al. 2024; Rasolozaka et al. in revision; Rabemananjara et al. 2025); and (5) active fire detection and management, including the use of satellite monitoring and community‐based fire surveillance programs, to enable rapid response to fire events before they reach ecologically damaging severity and extension. Improving fire detection through satellite data and community‐based management, alongside broader socioeconomic support for local communities, will be essential for achieving long‐term coexistence between lemurs and people in this landscape. Finally, (6) integrated conservation strategies that raise awareness for the destructive ecological consequences of uncontrolled fires will be essential for maintaining both lemur diversity and the ecological resilience of Madagascar's increasingly fire‐prone dry forest landscapes.

## Author Contributions

**Naina Ratsimba Rabemananjara:** investigation, methodology, formal analysis, writing – original draft, writing – review and editing, visualization. **Misa Rasolozaka:** investigation, writing – review and editing, methodology. **Marie Odile Ravolanirina:** investigation, writing – review and editing. **Rogula Marivola:** investigation, writing – review and editing. **Seheno Harilala Randriamiarantsoa:** investigation, writing – review and editing. **Romule Rakotondravony:** supervision, writing – review and editing. **Hanta Razafindraibe:** supervision, writing – review and editing. **Dominik Schüssler:** conceptualization, supervision, writing – review and editing, methodology, validation. **Ute Radespiel:** conceptualization, funding acquisition, writing – original draft, methodology, validation, project administration, data curation, supervision, resources, writing – review and editing.

## Supporting information

Supplement File

## Data Availability

The data that support the findings of this study are available from the corresponding authors upon reasonable request.
