# Supplementary material for: Influence of Vegetation Structure on Lemur Recolonization of Post‐Fire Habitats in Northwestern Madagascar
Source: Am J Primatol. 2026 Jul 5;88(7):e70186. doi: 10.1002/ajp.70186 (PMC13334197; doi:10.1002/ajp.70186)
Supplement: Supplementary file 1 — Supplement File [file AJP-88-e70186-s001.docx]

**Influence of vegetation structure on lemur recolonization of post-fire habitats in northwestern Madagascar**

Naina Ratsimba Rabemananjara^1^*, Misa Rasolozaka^1^, Marie Odile Ravolanirina^2^, Rogula Marivola^2^, Seheno Harilala Randriamiarantsoa^3^, Romule Rakotondravony^2^, Hanta Razafindraibe^3^, Dominik Schüßler^4^, Ute Radespiel^1^*

^1^Institute of Zoology, University of Veterinary Medicine Hannover, Hannover, Germany

^2^Mention Sciences de la Vie et de l’Environnement, Faculté des Sciences, de Technologies et de l’Environnement, University of Mahajanga, Mahajanga, Madagascar^.^

^3^Mention of Zoology and Animal Biodiversity, Faculté des Sciences, University of Antananarivo, Ambohitsaina, Madagascar

^4^Institute of Biology and Chemistry, University of Hildesheim, Hildesheim, Germany

***** Corresponding authors:** Ute Radespiel**,** Naina Ratsimba Rabemananjara

**Corresponding author emails:** ute.radespiel@tiho-hannover.de; indri.naina@gmail.com

**Supplementary Material**

**Supplementary Figures**

***
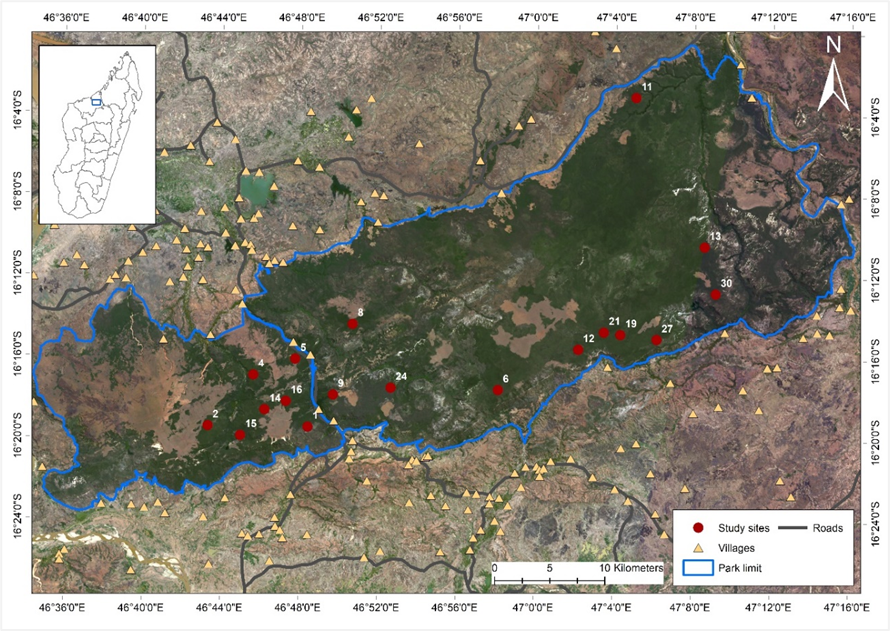
***

**FIGURE S1** Map of Ankarafantsika National Park located in northwestern Madagascar (inlay map) with the 18 study sites selected during prior remote sensing work (Source: Rabemananjara et al., 2025)

**
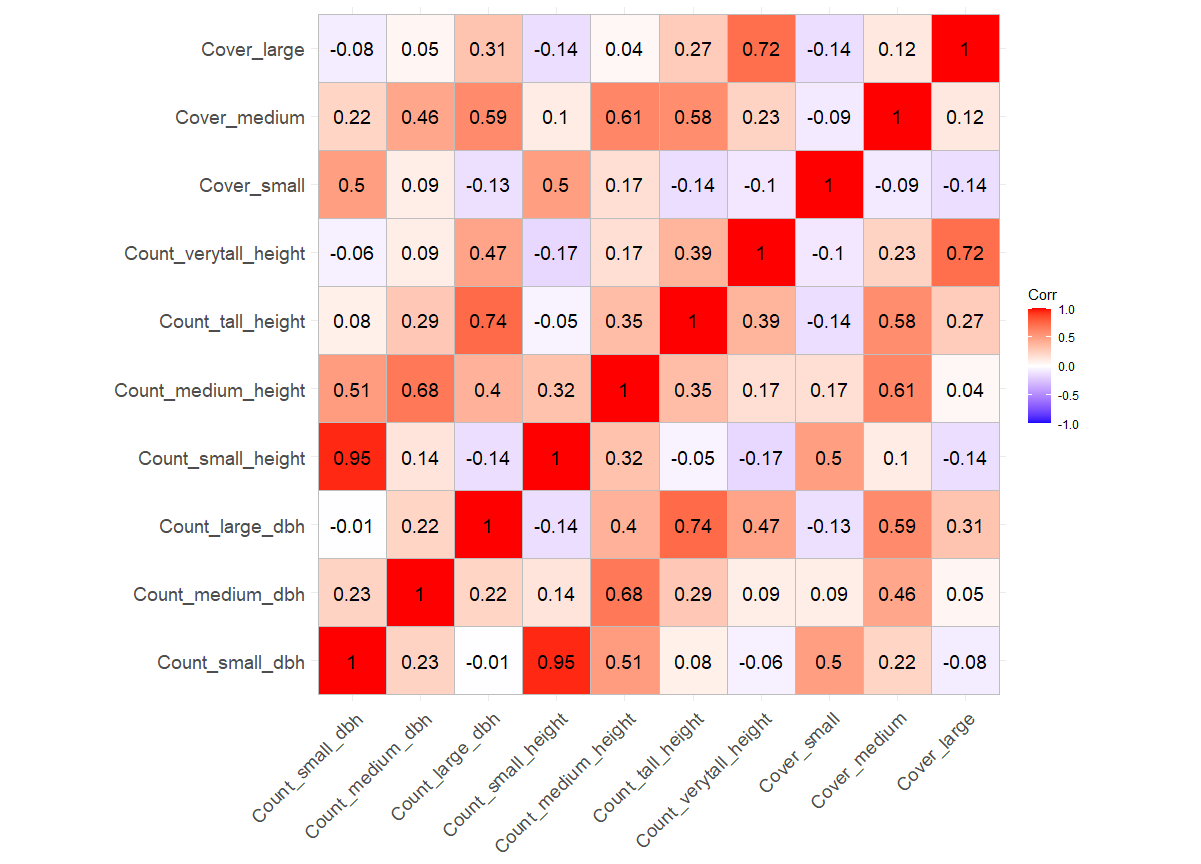
**

**FIGURE S2** Correlation heatmap of vegetation structural variables (DBH class, Height class, and forest strata cover class). Correlated variables with absolute values ≥ 0.7 were not included for PCA.


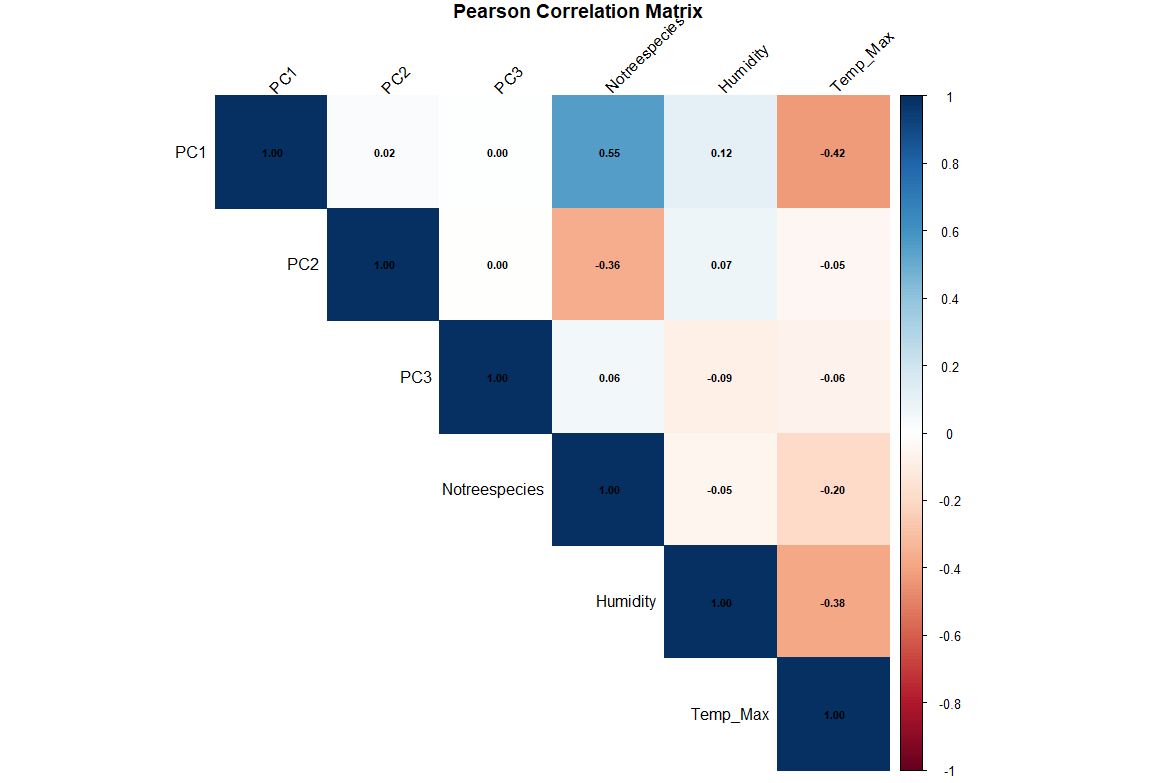


**FIGURE S3** Correlation heatmap (Pearson correlation coefficient) between three vegetation structural variables from the PCA, woody species richness, humidity, and maximum temperature.


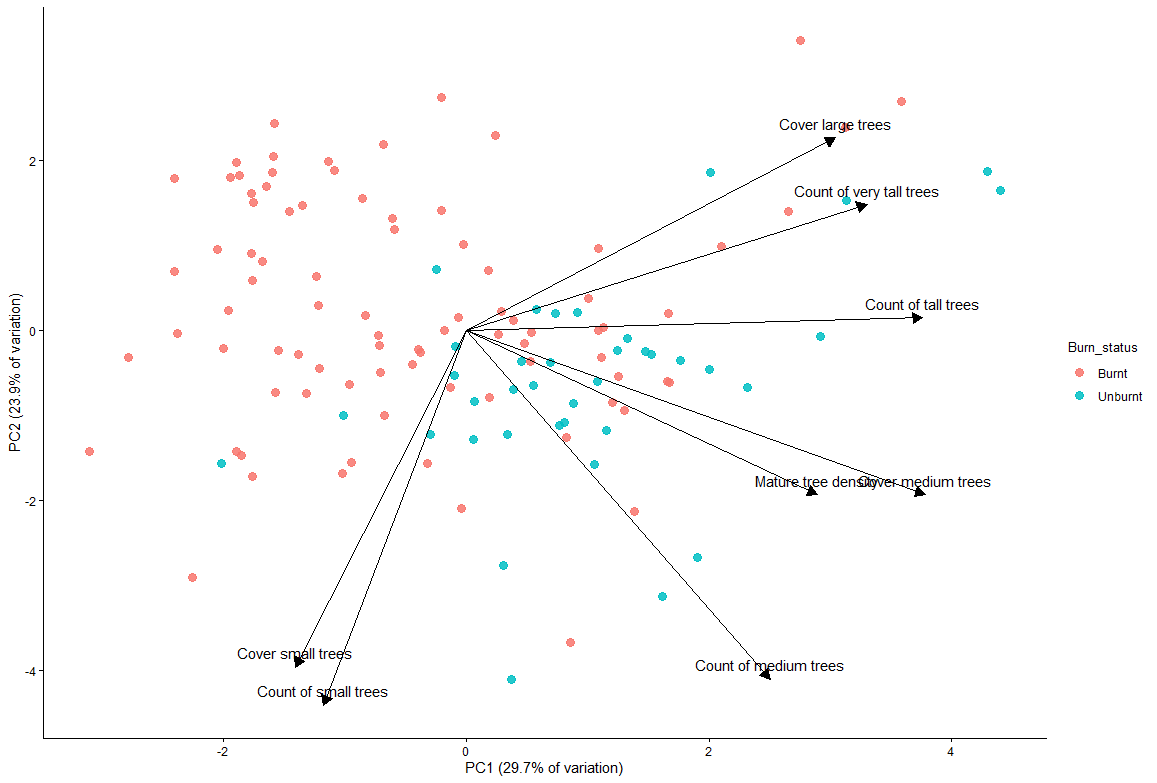


**FIGURE S4** Principal Component Analysis (PCA) biplot showing the separation between burnt plots (orange) and unburnt plots (green) along PC1 (29.7% variance explained) and PC2 (23.9% variance explained), together explaining 53.6% of total variance. PC1 represents a gradient of overall forest structural complexity, with plots scoring high on positive PC1 characterized by higher tall and very tall Woody counts, higher medium and upper stratum cover, and increased Woody density. PC2 depicts understory structure, with negative scores associated with denser small and medium tree counts and larger lower stratum cover, and positive scores reflecting more open understory conditions with greater upper canopy dominance. Three hierarchical clusters are shown by dashed ellipses:


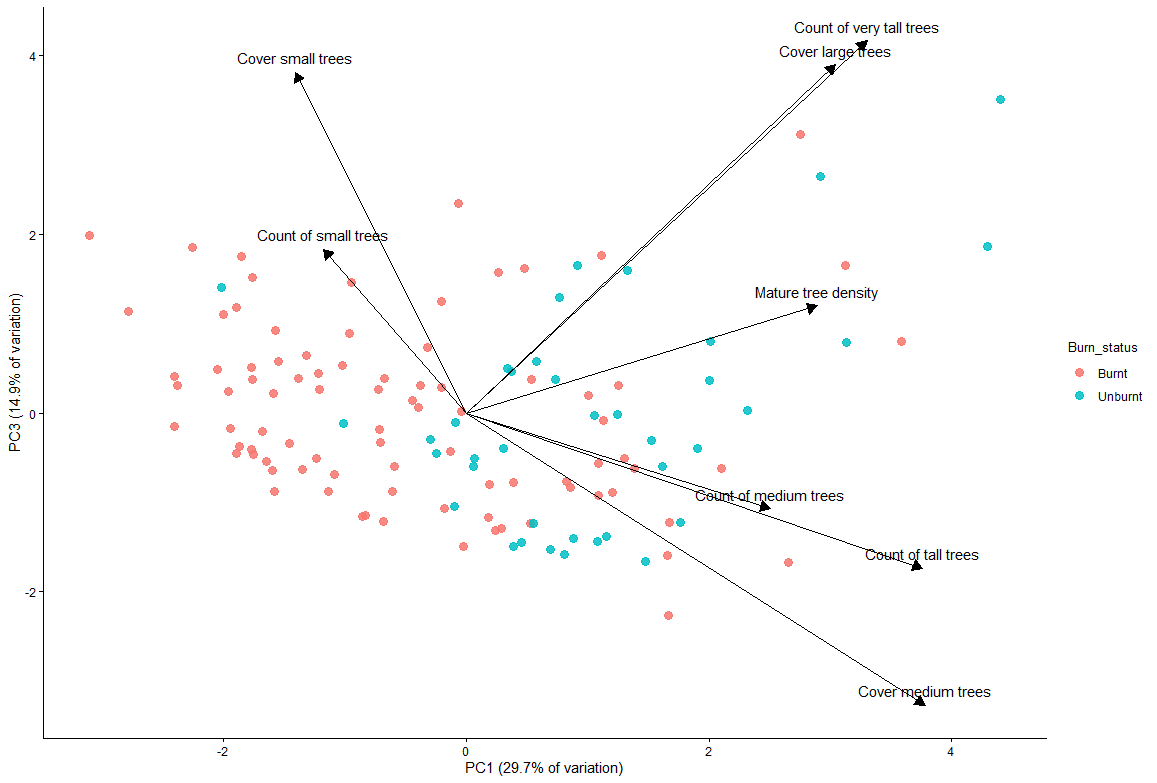


**FIGURE S5** Principal Component Analysis (PCA) biplot showing the separation between burnt plots (orange) and unburnt plots (green) along PC1 (29.7% variance explained) and PC3 (14.9% variance explained), explaining 44.6% of total variance combined. PC1 reflects a gradient of overall forest structural complexity, increasing with tall tree counts and upper stratum cover. PC3 contrasts plots with well-developed upper canopy, very tall trees, and higher lower stratum cover (positive scores) against those dominated by medium-sized trees and cover (negative scores), reflecting a two-layered forest structure with a tall canopy and a well-developed shrub layer.


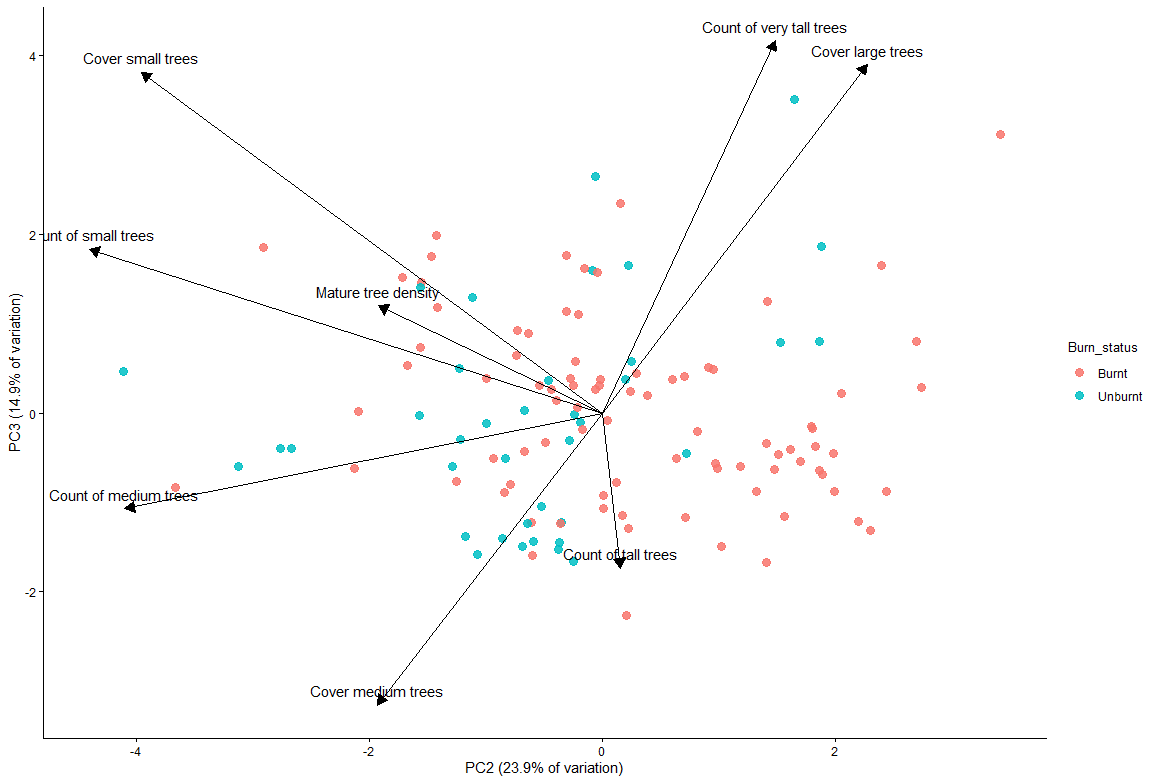


**FIGURE S6** Principal Component Analysis (PCA) biplot showing the separation between burnt plots (orange) and unburnt plots (green) along PC2 (23.9% variance explained) and PC3 (14.9% variance explained), explaining 38.8% of total variance combined. PC2 contrasts plots with denser small and medium tree counts and greater lower stratum cover (negative scores). PC3 contrasts plots with well-developed upper canopy, very tall trees, and greater lower stratum cover (positive scores) against those dominated by medium-sized trees and cover (negative scores), reflecting a two-layered forest structure.


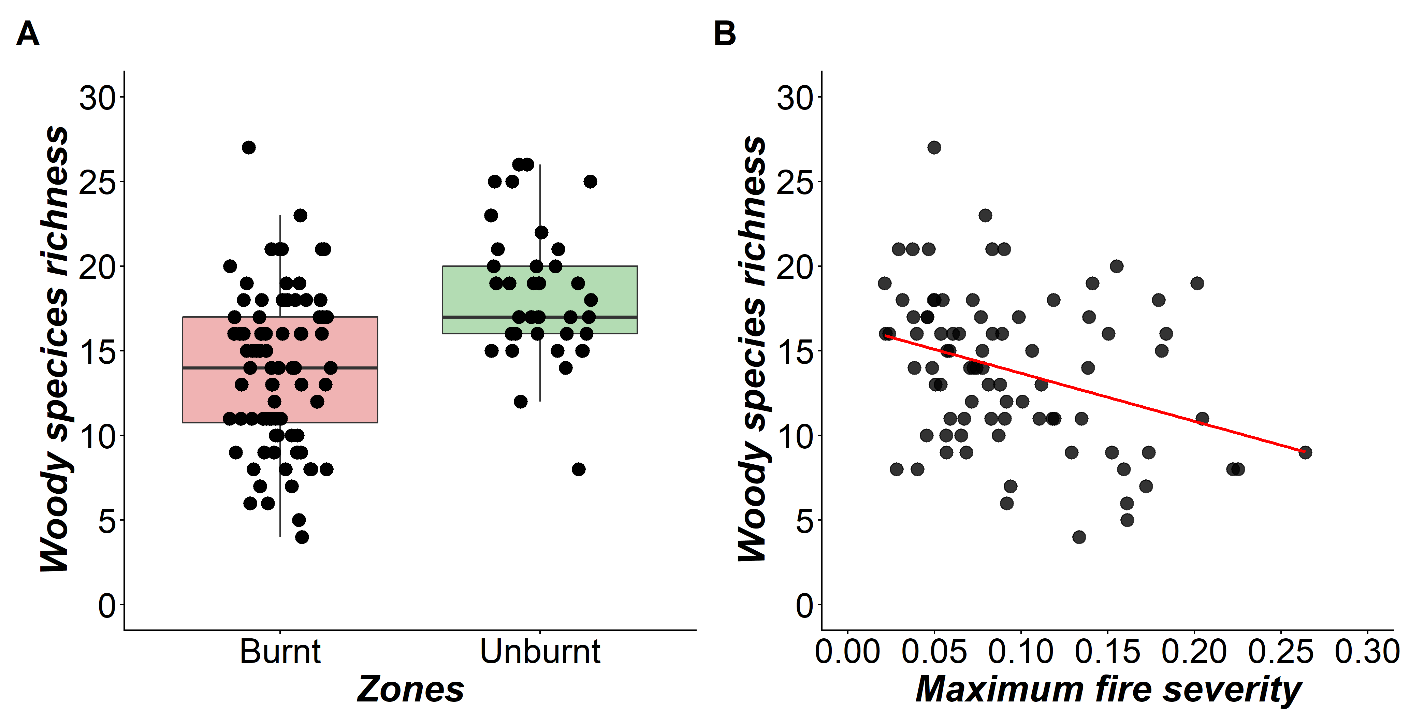


**FIGURE S7** Relationships between woody species richness and (A) different fire zones (unburnt vs. burnt) and (B) maximum fire severity. The box plot on the left shows the medians and interquartile ranges with individual values being added as jitter. The scatter plot on the right shows individual data points as black dots together with a fitted regression line.


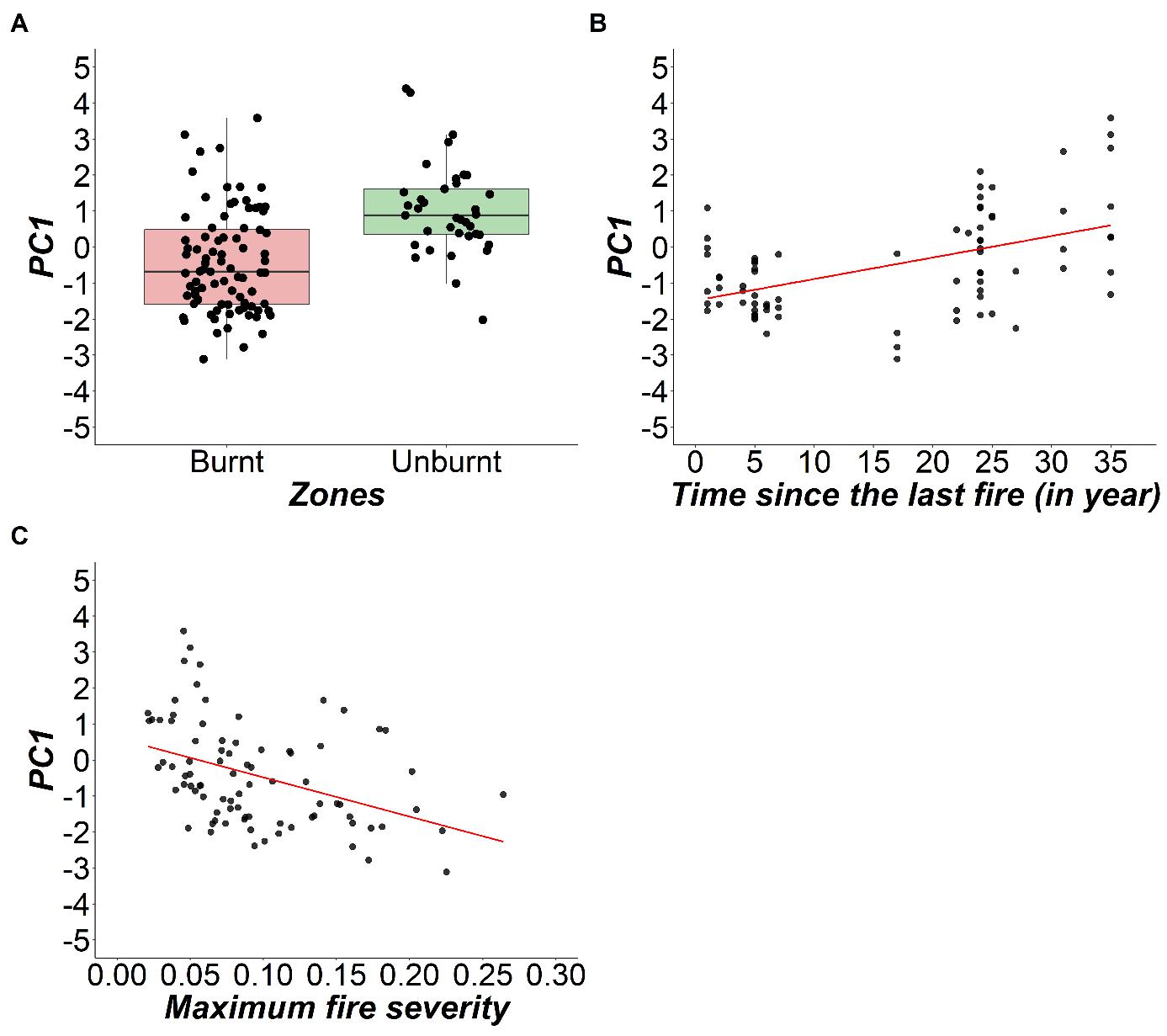


**FIGURE S8** Relationships between PC1 (overstory structural complexity and closure) and influential fire variables. (A) Difference in PC1 scores between fire zones (unburnt vs. burnt), illustrated by box plot showing the medians and interquartile ranges; individual datapoints are shown as jitter. (B, C) Relationship between PC1 scores and the time since the last fire (B) and maximum fire severity (C). Individual data points are shown together with fitted regression line.

**
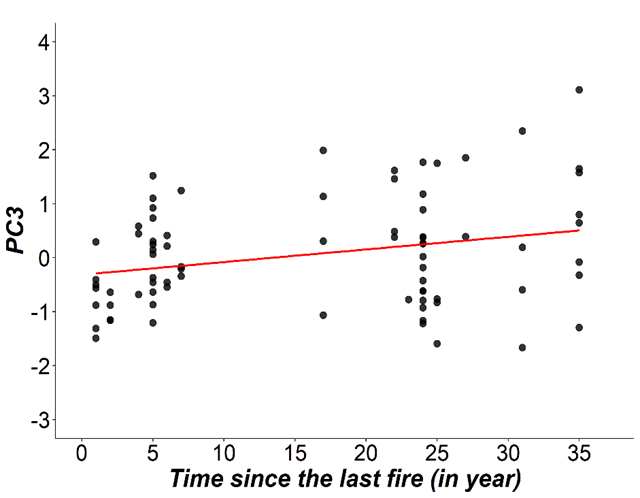
**

**FIGURE S9** Relationship between PC3 and the time since the last fire. Individual datapoints are shown in black dots together with a fitted regression line.

**Supplementary Tables**

**TABLE S1** Comparative ecological characteristics of lemur species occurring in Ankarafantsika National Park (source: Mittermeier et al., 2023). Body size is presented comparatively and reflects interspecific differences among the lemur species recorded in ANP

| Common name | Scientific name | Family | Body size | Locomotion | Feeding guild | Group size | Home range | Distribution/ habitat |
| --- | --- | --- | --- | --- | --- | --- | --- | --- |
| Coquerel’s sifaka | *Propithecus coquereli* | Indriidae | Large (~ 4 kg) | Vertical clinging and leaping | Folivore | 3-10 individuals | 4-9 ha | Northwestern Madagascar; dry forests |
| Mongoose lemur | *Eulemur mongoz* | Lemuridae | Large (~1.5kg) | Arboreal quadrupedal | Frugivore/ folivore/ nectar | 3-4 individuals | ~ 3 ha | Northwestern Madagascar; dry forests; introduced in Comoros |
| Common brown lemur | *Eulemur fulvus* | Lemuridae | Large (~2kg) | Arboreal quadrupedal | Frugivore/  folivore/ flowers | 3-12 individuals | 7-20 ha | Eastern rainy forest; northwestern dry forests of Madagascar; introduced in Comoros |
| Western woolly lemur | *Avahi occidentalis* | Indriidae | Medium-sized (~0.9kg) | Vertical clinging and leaping | Folivore | 1-5 individuals | 1-2 ha | Northwestern Madagascar; dry forests |
| Milne-Edwards’ sportive lemur | *Lepilemur edwardsi* | Lepilemuridae | Medium-sized (~1kg) | Vertical clinging and leaping | Folivore | Solitary but sleep together with up to 3 individuals | ~1.1 ha | Northwestern Madagascar; dry forests |
| Western fat-tailed dwarf lemur | *Cheirogaleus medius* | Cheirogaleidae | Small (~250g) | Arboreal quadrupedal | Primarily frugivore with some gums, insects and small vertebrates | 2 to 4 individuals | 1-2 ha | Southeastern rainy forest; western dry forest of Madagascar |
| Golden-brown mouse lemur | *Microcebus ravelobensis* | Cheirogaleidae | Small (~70g) | Arboreal quadrupedal | Omnivore (fruits, some gums, insects and insect secretion and small vertebrates) | Solitary but sleep in small social groups | 0.4-0.8 ha | Endemic to northwestern Madagascar; dry forests |
| Gray mouse lemur | *Microcebus murinus* | Cheirogaleidae | Small (~60g) | Arboreal quadrupedal | Omnivore (fruits, some gums, insects and insect secretions and small vertebrates) | Solitary but sleep in small social groups | 0.5-5 ha | Southeastern rainy forest; western dry forest of Madagascar |

**TABLE S2** Site and transect characteristics used in the study, with fire history ranging from 1988 to 2022. Columns from left to right: Site refers to the unique identifier for each sampling location; Years of fire lists all recorded fire events for each site (NA indicates data not available); Partition number is the number assigned to each partition within a transect; Length (meters) indicates the total length of each partition in meters; Transect zones describe the fire status of the transect zone (for example, unburnt); Transect partition provides the spatial code used during GPS flagging for the specific segment of the transect where the prefix indicates fire status: TLU for transect line unburnt, TLB for transect line burnt, and TLT for transect line transition.(for example: TLU000 to TLU500 refers to 0 – 500 m on the unburnt transect line in a given site); Time since the last fire represents the year between the last fire occurred and the year of site were visited; Maximum fire severity represent the highest value of dNBR among multiple fires in each partition.

| Site | Years of fire | Partition Number | Length (m) | Transect zones | Transect partition | Time since last fire (Year) | Maximum fire severity |
| --- | --- | --- | --- | --- | --- | --- | --- |
| 1 | NA | 1 | 500 | Unburnt | TLU000-TLU500 | NA | NA |
| 1 | 2021 | 2 | 200 | Burnt | TLT000-TLT200 | 1 | 0.073 |
| 1 | 2021 | 3 | 500 | Burnt | TLB000-TLB500 | 1 | 0.1144 |
| 9 | NA | 1 | 500 | Unburnt | TLU000-TLU500 | NA | NA |
| 9 | 2000 | 2 | 200 | Burnt | TLT000-TLT200 | 22 | 0.0869 |
| 9 | 2000 | 3 | 500 | Burnt | TLB000-TLB500 | 22 | 0.0504 |
| 4 | 1987, 1989, 1992, 1995, 1998 and 1999 | 1 | 110 | Burnt | TLU000-TLU110 | 23 | 0.0881 |
| 4 | 1987, 1992, 1995, 1998 and 1999 | 2 | 100 | Burnt | TLU120-TLU220 | 23 | 0.0657 |
| 4 | 1987, 1992, 1995 and 1998 | 3 | 140 | Burnt | TLU230-TLU360 | 24 | 0.0483 |
| 4 | 1987, 1992 and 1995 | 4 | 150 | Burnt | TLU370-TLU500 | 27 | 0.0528 |
| 4 | 1987, 1992, 1995, 1999 and 2017 | 5 | 200 | Burnt | TLT000-TLT200 | 5 | 0.0658 |
| 4 | 1987, 1992, 1995, 1998, 1999 and 2017 | 6 | 50 | Burnt | TLB000-TLB050 | 5 | 0.0948 |
| 4 | 1987, 1992, 1995, 1999 and 2017 | 7 | 450 | Burnt | TLB060-TLB500 | 5 | 0.0896 |
| 5 | 1988, 1998 and 2017 | 1 | 60 | Burnt | TLU000-TLU060 | 5 | 0.0638 |
| 5 | 1988 and 1998 | 2 | 240 | Burnt | TLU070-TLU300 | 24 | 0.0807 |
| 5 | 1998 and 2017 | 3 | 200 | Burnt | TLT000-TLT200 | 5 | 0.0852 |
| 5 | 1988, 1998 and 2017 | 4 | 30 | Burnt | TLB000-TLB030 | 5 | 0.051 |
| 5 | 1998 and 2017 | 5 | 470 | Burnt | TLB040-TLB500 | 5 | 0.0971 |
| 8 | NA | 1 | 500 | Unburnt | TLU000-TLU500 | NA | NA |
| 8 | 1998, 2002, 2006, 2010 and 2017 | 2 | 200 | Burnt | TLT000-TLT200 | 5 | 0.156 |
| 8 | 1998, 2002, 2006, 2010 and 2017 | 3 | 200 | Burnt | TLB000-TLB200 | 5 | 0.1966 |
| 8 | 1998, 2002, 2006 and 2017 | 4 | 300 | Burnt | TLB210-TLB500 | 5 | 0.1323 |
| 11 | NA | 1 | 500 | Unburnt | TLU000-TLU500 | NA | NA |
| 11 | 1995 and 1998 | 2 | 200 | Burnt | TLT000-TLT200 | 24 | 0.0689 |
| 11 | 1995 and 1998 | 3 | 500 | Burnt | TLB000-TLB500 | 24 | 0.1154 |
| 15 | 1988 | 1 | 500 | Burnt | TLU000-TLU500 | 35 | 0.0428 |
| 15 | 1988 and 1992 | 2 | 200 | Burnt | TLT000-TLT200 | 31 | 0.0633 |
| 15 | 1988 and 1992 | 3 | 500 | Burnt | TLB000-TLB500 | 31 | 0.0407 |
| 6 | NA | 1 | 500 | Unburnt | TLU000-TLU500 | NA | NA |
| 6 | 1998 and 2018 | 2 | 200 | Burnt | TLT000-TLT200 | 5 | 0.0367 |
| 6 | 1998 and 2018 | 3 | 330 | Burnt | TLB000-TLB330 | 5 | 0.0852 |
| 6 | 1986, 1998 and 2018 | 4 | 170 | Burnt | TLB340-TLB500 | 5 | 0.1345 |
| 12 | NA | 1 | 160 | Unburnt | TLU350-TLU500 | NA | NA |
| 12 | 1986 | 2 | 340 | Burnt | TLU00-TLU340 | >35 | NA |
| 12 | 1986 and 1998 | 3 | 200 | Burnt | TLT000-TLT200 | 25 | 0.1565 |
| 12 | 1986 and 1998 | 4 | 500 | Burnt | TLB000-TLB500 | 25 | 0.1402 |
| 2 | 1988 and 1999 | 1 | 500 | Burnt | TLU000-TLU500 | 24 | 0.0694 |
| 2 | 1988 and 1999 | 2 | 200 | Burnt | TLT000-TLT200 | 24 | 0.1504 |
| 2 | 1988 and 1999 | 3 | 500 | Burnt | TLB000-TLB500 | 24 | 0.1718 |
| 19 | > 1986 | 1 | 500 | Burnt | TLU000-TLU500 | NA | NA |
| 19 | NA | 2 | 200 | Unburnt | TLT000-TLT500 | NA | NA |
| 19 | NA | 3 | 500 | Unburnt | TLB000-TLB500 | >35 | NA |
| 21 | NA | 1 | 500 | Unburnt | TLU000-TLU500 | NA | NA |
| 21 | 2016 | 2 | 200 | Burnt | TLT000-TLT200 | 7 | 0.0912 |
| 21 | 2016 | 3 | 500 | Burnt | TLB000-TLB500 | 7 | 0.057 |
| 30 | NA | 1 | 500 | Unburnt | TLU000-TLU500 | NA | NA |
| 30 | 2017 | 2 | 200 | Burnt | TLT000-TLT200 | 6 | 0.1446 |
| 30 | 2017 | 3 | 500 | Burnt | TLB000-TLB500 | 6 | 0.1167 |
| 24 | NA | 1 | 500 | Unburnt | TLU000-TLU500 | NA | NA |
| 24 | 1998 and 2022 | 2 | 200 | Burnt | TLT000-TLT200 | 1 | 0.07 |
| 24 | 1998 and 2022 | 3 | 500 | Burnt | TLB000-TLB500 | 1 | 0.116 |
| 16 | NA | 1 | 500 | Unburnt | TLU000-TLU500 | NA | NA |
| 16 | 1988 | 2 | 200 | Burnt | TLT000-TLT200 | 35 | 0.0657 |
| 16 | 1988 | 3 | 500 | Burnt | TLB000-TLB500 | 35 | 0.0926 |
| 14 | NA | 1 | 500 | Unburnt | TLU000-TLU500 | NA | NA |
| 14 | 1988 | 2 | 90 | Burnt | TLT000-TLT090 | 35 | 0.0022 |
| 14 | 1988 and 2019 | 3 | 110 | Burnt | TLT100-TLT200 | 4 | 0.0191 |
| 14 | 1988 | 4 | 120 | Burnt | TLB000-TLB120 | 35 | 0.0564 |
| 14 | 1988, 1996 and 2019 | 6 | 250 | Burnt | TLB130-TLB380 | 27 | 0.0857 |
| 14 | 1988 and 1996 | 5 | 110 | Burnt | TLB390-TLB500 | 4 | 0.0739 |
| 13 | 1999 | 1 | 500 | Burnt | TLU000-TLU500 | 24 | 0.0298 |
| 13 | 1999 and 2006 | 2 | 200 | Burnt | TLT000-TLT200 | 17 | 0.1301 |
| 13 | 1999 and 2006 | 3 | 500 | Burnt | TLB000-TLB500 | 17 | 0.1703 |
| 27 | NA | 1 | 480 | Unburnt | TLU000-TLU480 | NA | NA |
| 27 | 2021 | 2 | 100 | Burnt | TLT000-TLT100 | 2 | 0.1243 |
| 27 | 2021 | 3 | 500 | Burnt | TLB000-TLB500 | 2 | 0.1058 |

**TABLE S3**. Abiotic conditions recorded across phenology plots in burnt and unburnt transect zones across all study sites. Mean maximum temperature (°C) and mean relative humidity (%) measured in phenology plots located within burnt and unburnt transect zones during the site visit. Abiotic data was collected hourly using automated data loggers (GSP-6, Elitech), and values shown represent averages calculated across the sampling period for each floristic plot. NA: not available

| Site | Floristic plot | Transect zone | Month of  visit | Mean of maximum temperature (°C) | Mean of humidity (%) | Woody species richness per plot |
| --- | --- | --- | --- | --- | --- | --- |
| 1 | 1U | Unburnt | September | 34.3 | 52.12 | 25 |
| 1 | 2U | Unburnt | September | 36.9 | 40.75 | 25 |
| 1 | 3U | Unburnt | September | 35 | 59.44 | 26 |
| 1 | 4B | Burnt | September | 35.3 | 60.89 | 21 |
| 1 | 5B | Burnt | September | 34.1 | 55.68 | 16 |
| 1 | 6B | Burnt | September | 40.5 | 55.75 | 8 |
| 1 | 7T | Burnt | September | 39.3 | 50.68 | 6 |
| 9 | 1U | Unburnt | September | 39.2 | 52.27 | 18 |
| 9 | 2U | Unburnt | September | 39.5 | 59.48 | 20 |
| 9 | 3U | Unburnt | September | 39.5 | 59.36 | 26 |
| 9 | 4B | Burnt | September | 39 | 54.14 | 16 |
| 9 | 5B | Burnt | September | 40.3 | 48.05 | 11 |
| 9 | 6B | Burnt | September | 37 | 60.76 | 14 |
| 9 | 7T | Burnt | September | 36.7 | 60.7 | 13 |
| 4 | 1U | Burnt | October | 36.6 | 74.48 | 17 |
| 4 | 2U | Burnt | October | 39 | 77.21 | 13 |
| 4 | 3U | Burnt | October | 36.2 | 72.45 | 17 |
| 4 | 4B | Burnt | October | 36.5 | 56.78 | 13 |
| 4 | 5B | Burnt | October | 37.8 | 69.46 | 16 |
| 4 | 6B | Burnt | October | 39.7 | 60.47 | 11 |
| 4 | 7T | Burnt | October | 40.4 | 65.4 | 9 |
| 8 | 1U | Unburnt | October | 36.1 | 71.36 | 17 |
| 8 | 2U | Unburnt | October | 37.9 | 69.76 | 19 |
| 8 | 3U | Unburnt | October | 36 | 76.5 | 21 |
| 8 | 4B | Burnt | October | 38.1 | 72.2 | 8 |
| 8 | 5B | Burnt | October | 39.1 | 70.46 | 11 |
| 8 | 6B | Burnt | October | 38.3 | 72.8 | 14 |
| 8 | 7T | Burnt | October | 33.1 | 87.7 | 19 |
| 5 | 1U | Burnt | November | 36.1 | 91.82 | 21 |
| 5 | 2U | Burnt | November | 36.5 | 83.94 | 18 |
| 5 | 4B | Burnt | November | 39.9 | 87.2 | 23 |
| 5 | 5B | Burnt | November | 41.5 | 83.2 | 11 |
| 5 | 6B | Burnt | November | 39 | 85.2 | 15 |
| 5 | 7T | Burnt | November | 38.3 | 81.86 | 21 |
| 11 | 1U | Unburnt | November | 35 | 80.62 | 15 |
| 11 | 2U | Unburnt | November | 34.1 | NA | 18 |
| 11 | 3U | Unburnt | November | 38.9 | 98.56 | 15 |
| 11 | 4B | Burnt | November | 37.9 | 88.94 | 9 |
| 11 | 5B | Burnt | November | 35.4 | 93.39 | 10 |
| 11 | 6B | Burnt | November | 37.5 | 89.96 | 9 |
| 11 | 7T | Burnt | November | 44.6 | 92.75 | 17 |
| 15 | 1U | Burnt | May | 29.3 | 98.27 | 18 |
| 15 | 2U | Burnt | May | 28.2 | 95.3 | 10 |
| 15 | 3U | Burnt | May | 28 | 93.89 | 17 |
| 15 | 4B | Burnt | May | 31.7 | 95.04 | 18 |
| 15 | 5B | Burnt | May | 30.9 | 94.14 | 15 |
| 15 | 6B | Burnt | May | 30.6 | 96.96 | 9 |
| 15 | 7T | Burnt | May | 34.3 | 84.07 | 15 |
| 6 | 1U | Unburnt | May | 31.3 | 77.57 | 25 |
| 6 | 2U | Unburnt | May | 31.4 | 75.88 | 19 |
| 6 | 3U | Unburnt | May | 31 | 76.04 | 23 |
| 6 | 4B | Burnt | May | 31.4 | 78.07 | 27 |
| 6 | 5B | Burnt | May | NA | 79.33 | 14 |
| 6 | 6B | Burnt | May | NA | 75.48 | 4 |
| 6 | 7T | Burnt | May | 32.1 | 80.9 | 14 |
| 12 | 1U | Burnt | June | 29.9 | 86.71 | 21 |
| 12 | 2U | Burnt | June | 30.2 | 84.82 | 14 |
| 12 | 3U | Unburnt | June | 29.6 | 86.04 | 19 |
| 12 | 4B | Burnt | June | 28.8 | 88.55 | 19 |
| 12 | 5B | Burnt | June | 30.1 | 85.77 | 15 |
| 12 | 6B | Burnt | June | 30.4 | 88.01 | 18 |
| 12 | 7T | Burnt | June | 29.7 | 87.6 | 16 |
| 2 | 1U | Burnt | July | 32.6 | 84.6 | 20 |
| 2 | 2U | Burnt | July | 30.5 | 84.86 | 16 |
| 2 | 3U | Burnt | July | 31.9 | 85.35 | 18 |
| 2 | 4B | Burnt | July | 29.8 | 83.48 | 16 |
| 2 | 5B | Burnt | July | 30.7 | 85.55 | 11 |
| 2 | 6B | Burnt | July | 29.8 | 83.28 | 18 |
| 2 | 7T | Burnt | July | 30.7 | 83.89 | 16 |
| 19 | 1U | Burnt | July | 31.8 | 79.71 | 19 |
| 19 | 2U | Burnt | July | 31.2 | 76.62 | 16 |
| 19 | 3U | Burnt | July | 31 | 71.16 | 16 |
| 19 | 4B | Unburnt | July | 32 | 78.69 | 16 |
| 19 | 5B | Unburnt | July | 32 | 72.5 | 8 |
| 19 | 6B | Unburnt | July | 32.5 | 75.82 | 16 |
| 19 | 7T | Unburnt | July | 33 | 76.51 | 16 |
| 21 | 1U | Unburnt | August | 31.3 | 74.08 | 17 |
| 21 | 2U | Unburnt | August | 31.9 | 75.37 | 15 |
| 21 | 3U | Unburnt | August | 32.5 | 72.76 | 12 |
| 21 | 4B | Burnt | August | 32.5 | 75.71 | 8 |
| 21 | 5B | Burnt | August | 31.7 | 78.3 | 11 |
| 21 | 6B | Burnt | August | 34.5 | 76.72 | 9 |
| 21 | 7T | Burnt | August | 32 | 74.8 | 12 |
| 30 | 1U | Unburnt | August | 33.8 | 77.78 | 17 |
| 30 | 2U | Unburnt | August | 36 | 79.55 | 17 |
| 30 | 3U | Unburnt | August | 34.3 | 80.58 | 19 |
| 30 | 4B | Burnt | August | 35.2 | 79.34 | 4 |
| 30 | 5B | Burnt | August | 34.5 | 76.78 | 10 |
| 30 | 6B | Burnt | August | 34.8 | 73.31 | 6 |
| 30 | 7T | Burnt | August | 35.5 | 76.68 | 5 |
| 24 | 1U | Unburnt | September | 41.6 | 73.43 | 17 |
| 24 | 2U | Unburnt | September | 35.8 | 76.26 | 20 |
| 24 | 3U | Unburnt | September | 35 | 73.81 | 19 |
| 24 | 4B | Burnt | September | 35.7 | 76.87 | 14 |
| 24 | 5B | Burnt | September | 36.6 | 75.42 | 9 |
| 24 | 6B | Burnt | September | 35.6 | 74.34 | 11 |
| 24 | 7T | Burnt | September | 35.2 | 76.26 | 10 |
| 16 | 1U | Unburnt | September | 37.7 | 78.17 | 14 |
| 16 | 2U | Unburnt | September | 38.4 | 74.14 | 20 |
| 16 | 3U | Unburnt | September | 37.4 | 68.66 | 22 |
| 16 | 4B | Burnt | September | 36.6 | 73.32 | 11 |
| 16 | 5B | Burnt | September | 38.2 | 70.61 | 15 |
| 16 | 6B | Burnt | September | 36 | 60.36 | 17 |
| 16 | 7T | Burnt | September | 37.4 | 60.31 | 12 |
| 14 | 1U | Unburnt | October | 38 | 82.5 | 15 |
| 14 | 2U | Unburnt | October | 38.2 | 72.88 | 15 |
| 14 | 3U | Unburnt | October | 37.7 | 79.4 | 16 |
| 14 | 4B | Burnt | October | 37.5 | 70.04 | 16 |
| 14 | 5B | Burnt | October | 38.6 | 79.33 | 14 |
| 14 | 6B | Burnt | October | 38.7 | 63.06 | 12 |
| 14 | 7T | Burnt | October | 42.4 | 79.33 | 11 |
| 13 | 1U | Burnt | October | 36.9 | 87.31 | 16 |
| 13 | 2U | Burnt | October | 37.4 | 81.23 | 21 |
| 13 | 3U | Burnt | October | 38.5 | 81.87 | 18 |
| 13 | 4B | Burnt | October | 43.9 | 76.83 | 7 |
| 13 | 5B | Burnt | October | 41.2 | 83.63 | 7 |
| 13 | 6B | Burnt | October | 38.1 | 81.65 | 8 |
| 13 | 7T | Burnt | October | 37.4 | 65.54 | 17 |
| 27 | 1U | Unburnt | November | 35.4 | 81.06 | 16 |
| 27 | 2U | Unburnt | November | 36.1 | 88.62 | 15 |
| 27 | 3U | Unburnt | November | 37.5 | 76.41 | 21 |
| 27 | 4B | Burnt | November | 37 | 81.24 | 13 |
| 27 | 5B | Burnt | November | 35.9 | 81.86 | 14 |
| 27 | 6B | Burnt | November | 36 | 83.62 | 13 |
| 27 | 7T | Burnt | November | 39.9 | 77.36 | 8 |

**TABLE S4** Summary of the principal component analysis (PCA) for vegetation-structure variables. Only components with eigenvalues greater than 1 were retained for subsequent analyses. The table reports the eigenvalues, the proportion of variance explained, and the cumulative proportion of variance explained by each principal component. The first three components together account for 68.5% of the total variance. Loadings of the vegetation-structure variables on each principal component are also presented. Variables with loadings > 0.3 were considered to contribute meaningfully to a component and were highlighted in grey for PC1–PC3, which were included in further analyses. PC1 reflects overstory complexity and canopy closure. PC2 primarily describes openness in the two lower strata, with negative loadings for small trees. PC3 corresponds to habitats characterized by high forest cover above 10 m and high counts of very tall trees, combined with a dense understory but low cover in the medium stratum.

|  | PC1 | PC2 | PC3 | PC4 | PC5 | PC6 | PC7 | PC8 |
| --- | --- | --- | --- | --- | --- | --- | --- | --- |
| Eigenvalue | 1.541066 | 1.382385 | 1.093097 | 0.932705 | 0.782867 | 0.694038 | 0.543952 | 0.514399 |
| Proportion of explained variance | 0.29665 | 0.2387 | 0.14925 | 0.10866 | 0.07656 | 0.06017 | 0.03696 | 0.03305 |
| Cumulative Proportion | 0.29665 | 0.53535 | 0.6846 | 0.79327 | 0.86982 | 0.92999 | 0.96695 | 1 |
| Woody plant density | 0.354342204 | -0.235898451 | 0.146874254 | -0.708564149 | -0.344122725 | -0.2938651 | 0.142785583 | 0.264562758 |
| Count of small trees | -0.143940239 | -0.539336606 | 0.224224387 | 0.450535037 | 0.143702667 | -0.40612261 | 0.063719592 | 0.495472118 |
| Count of medium trees | 0.30668912 | -0.501985849 | -0.130911211 | -0.171784534 | 0.46922266 | 0.08094985 | -0.568967323 | -0.238448575 |
| Count of tall trees | 0.46094454 | 0.018803492 | -0.212752516 | 0.423291585 | -0.610448691 | 0.06759134 | -0.403710953 | 0.150128054 |
| Count of very tall trees | 0.40451384 | 0.181851619 | 0.510042755 | 0.047472787 | 0.280274922 | 0.54624921 | 0.067445588 | 0.39926197 |
| Cover of small stratum | -0.172569102 | -0.484720044 | 0.46650745 | 0.070433109 | -0.403611367 | 0.3683627 | 0.11356641 | -0.448537417 |
| Cover of medium stratum | 0.463313898 | -0.23650935 | -0.400173058 | 0.200846807 | 0.129916295 | 0.07716795 | 0.686362524 | -0.187080176 |
| Cover of tall stratum | 0.372992513 | 0.277784767 | 0.477324533 | 0.196762308 | 0.098879591 | -0.54548335 | -0.017492617 | -0.457736654 |

**TABLE S5** Model selection results showing the best-fitting models for different lemur species and lemur species richness, with associated model parameters and selection criteria. The table includes the species assessed, the component model representing the set of predictor variables, the degrees of freedom (df), the log-likelihood (logLik), the corrected Akaike Information Criterion (AICc) which balances fit and complexity and is corrected for small sample sizes with lower values representing better models, the delta (ΔAICc) which is the difference between a given model and the best-fitting model, and the Akaike weight which reflects the relative likelihood of a model being the best among the candidate models. Null: null model that does not contain any predictor variable. PC: principal component.

| Species | Component model | df | logLik | AICc | Delta (AICc) | Akaike Weight |
| --- | --- | --- | --- | --- | --- | --- |
| *Eulemur fulvus* | Humidity + Woody species richness | 4 | -33.78 | 75.9 | 0.0 | 0.32 |
|  | Humidity + Woody species richness + PC1 | 5 | -33.0 | 76.53 | 0.63 | 0.24 |
|  | Humidity +Woody species richness + PC3 | 5 | -33.36 | 77.25 | 1.34 | 0.16 |
|  | Humidity + PC1 | 4 | -34.49 | 77.33 | 1.42 | 0.16 |
|  | Humidity + Woody species richness + PC2 | 5 | -33.68 | 77.88 | 1.98 | 0.12 |
| *Lepilemur edwardsi* | PC1 | 3 | -59.79 | 125.79 | 0.0 | 0.35 |
|  | Humidity + PC1 | 4 | -59.37 | 127.09 | 1.3 | 0.18 |
|  | PC1 + Maximum temperature | 4 | -59.42 | 127.19 | 1.39 | 0.17 |
|  | Woody species richness + PC1 | 4 | -59.49 | 127.32 | 1.52 | 0.16 |
|  | PC1 + PC3 | 4 | -59.67 | 127.69 | 1.9 | 0.13 |
| *Avahi occidentalis* | Woody species richness | 3 | -55.29 | 116.79 | 0.0 | 0.29 |
|  | Woody species richness + PC1 | 4 | -54.68 | 117.71 | 0.92 | 0.18 |
|  | Woody species richness + PC3 | 4 | -54.69 | 117.72 | 0.93 | 0.18 |
|  | Woody species richness + PC3 + PC1 | 5 | -54.01 | 118.54 | 1.75 | 0.12 |
|  | Woody species richness + PC2 | 4 | -55.12 | 118.59 | 1.8 | 0.12 |
|  | Humidity + Woody species richness | 4 | -55.15 | 118.64 | 1.85 | 0.11 |
| *Cheirogaleus medius* | Woody species richness | 3 | -37.2 | 80.72 | 0.0 | 0.15 |
|  | Humidity + Woody species richness | 4 | -36.16 | 80.84 | 0.13 | 0.14 |
|  | Woody species richness + PC2 | 4 | -36.38 | 81.28 | 0.56 | 0.11 |
|  | Humidity + Woody species richness + PC2 | 5 | -35.36 | 81.52 | 0.8 | 0.1 |
|  | PC2 + PC3 | 4 | -36.65 | 81.83 | 1.11 | 0.08 |
|  | PC2 | 3 | -37.79 | 81.88 | 1.17 | 0.08 |
|  | Woody species richness + PC3 | 4 | -36.69 | 81.9 | 1.19 | 0.08 |
|  | Woody species richness + PC2 + PC3 | 5 | -35.58 | 81.95 | 1.24 | 0.08 |
|  | Woody species richness + Maximum temperature | 4 | -36.85 | 82.23 | 1.51 | 0.07 |
|  | Humidity + Woody species richness + Maximum temperature | 5 | -35.82 | 82.44 | 1.73 | 0.06 |
|  | Humidity + Woody species richness + PC3 | 5 | -35.9 | 82.61 | 1.89 | 0.06 |
| *Microcebus ravelobensis* | Humidity + PC3 | 4 | -66.39 | 141.13 | 0.0 | 0.26 |
|  | Humidity + PC1 + PC3 | 5 | -65.68 | 141.88 | 0.74 | 0.18 |
|  | Humidity + PC2 + PC3 | 5 | -65.69 | 141.91 | 0.78 | 0.17 |
|  | Humidity + Woody species richness + PC3 | 5 | -65.86 | 142.25 | 1.12 | 0.15 |
|  | Humidity + PC1 + PC2 + PC3 | 6 | -64.86 | 142.45 | 1.32 | 0.13 |
|  | Humidity + PC3 + Maximum temperature | 5 | -66.08 | 142.68 | 1.55 | 0.12 |
| *Microcebus murinus* | Null | 2 | -81.84 | 167.78 | 0.0 | 0.25 |
|  | Woody species richness | 3 | -81.17 | 168.55 | 0.78 | 0.17 |
|  | Humidity | 3 | -81.5 | 169.2 | 1.42 | 0.12 |
|  | Maximum temperature | 3 | -81.5 | 169.2 | 1.43 | 0.12 |
|  | PC1 | 3 | -81.52 | 169.25 | 1.47 | 0.12 |
|  | PC2 | 3 | -81.68 | 169.56 | 1.78 | 0.1 |
|  | Humidity + Maximum temperature | 4 | -80.64 | 169.63 | 1.85 | 0.1 |
| Lemur species richness | Woody species richness + PC1 | 5 | -177.13 | 364.79 | 0.0 | 0.21 |
|  | Woody species richness | 4 | -178.27 | 364.89 | 0.1 | 0.2 |
|  | Humidity + Woody species richness | 5 | -177.69 | 365.9 | 1.11 | 0.12 |
|  | Humidity + Woody species richness + PC1 | 6 | -176.76 | 366.26 | 1.47 | 0.1 |
|  | Woody species richness + PC1 + PC2 | 6 | -176.82 | 366.38 | 1.59 | 0.1 |
|  | Woody species richness + PC1 + PC3 | 6 | -176.85 | 366.43 | 1.64 | 0.09 |
|  | Woody species richness + PC3 | 5 | -178.04 | 366.61 | 1.82 | 0.09 |
|  | Woody species richness + PC1 + Maximum temperature | 6 | -176.98 | 366.7 | 1.91 | 0.08 |

**TABLE S6** Model-averaged parameter estimates of predictors for the presence of *Eulemur fulvus*. Estimates represent the effect size of each predictor on the response variable. SE (Standard Error) indicates the precision of the estimate. The 95% Confidence Interval (95% CI) shows the range of values within which the true effect is likely to fall. Predictors are considered influential if the confidence interval does not include zero (respective results in bold). Relative Importance (RI) represents the sum of Akaike weights across all relevant models (from Table S2) in which the predictor appeared. Variables with an RI ≥ 0.8 are considered strongly supported (Burnham et al., 2011; Symonds & Moussalli, 2011).

| Species | Variables | Estimate | SE | 95% CI | Relative Importance (RI) |
| --- | --- | --- | --- | --- | --- |
| *Eulemur fulvus* | **Forest humidity** | **0.0754** | **0.0371** | **[0.0020, 0.1489]** | **1.0000** |
|  | **Woody species richness** | **0.1656** | **0.0834** | **[0.0005, 0.3308]** | **0.8419** |
|  | PC1 | 0.3269 | 0.2240 | [-0.1165, 0.7703] | 0.3934 |
|  | PC2 | 0.1060 | 0.2395 | [-0.3683, 0.5803] | 0.1198 |
|  | PC3 | 0.2446 | 0.2665 | [-0.2832, 0.7724] | 0.1645 |

**TABLE S7** Model-averaged parameter estimates of predictors for the presence of the medium-sized *Lepilemur edwardsi* and *Avahi occidentalis,* respectively. Estimates represent the effect size of each predictor on the response variable. SE (Standard Error) indicates the precision of the estimate. The 95% Confidence Interval (95% CI) shows the range of values within which the true effect is likely to fall. Predictors are considered influential if the confidence interval does not include zero (respective results in bold). Relative Importance (RI) represents the sum of Akaike weights across all relevant models (from Table S2) in which the predictor appeared. Variables with an RI ≥ 0.8 are considered strongly supported (Burnham et al., 2011; Symonds & Moussalli, 2011).

| Species | Variables | Estimate | SE | 95% CI | Relative Importance (RI) |
| --- | --- | --- | --- | --- | --- |
| *Lepilemur edwardsi* | Forest humidity | -0.0216 | 0.0233 | [-0.0677, 0.0245] | 0.1820 |
|  | Woody species richness | 0.0450 | 0.0573 | [-0.0686, 0.1585] | 0.1623 |
|  | Maximum Temperature | 0.0773 | 0.0837 | [-0.0884, 0.2430] | 0.1732 |
|  | **PC1** | **0.4378** | **0.1761** | **[0.0892, 0.7864]** | **1.0000** |
|  | PC3 | 0.1037 | 0.2094 | [-0.3111, 0.5185] | 0.1348 |
| *Avahi occidentalis* | Forest humidity | -0.0114 | 0.0211 | [-0.0532, 0.0304] | 0.1139 |
|  | **Woody species richness** | **0.1469** | **0.0611** | **[0.0261, 0.2676]** | **1.0000** |
|  | PC1 | -0.2261 | 0.2078 | [-0.6376, 0.1854] | 0.3012 |
|  | PC2 | -0.1072 | 0.1925 | [-0.4885, 0.2740] | 0.1168 |
|  | PC3 | -0.2506 | 0.2302 | [-0.7064, 0.2053] | 0.3007 |

**TABLE S8** Model-averaged parameter estimates of predictors for the presence of the small-sized *Cheirogaleus medius, Microcebus ravelobensis* and *M. murinus,* respectively. Estimates represent the effect size of each predictor on the response variable. SE (Standard Error) indicates the precision of the estimate. The 95% Confidence Interval (95% CI) shows the range of values within which the true effect is likely to fall. Predictors are considered influential if the confidence interval does not include zero (respective results in bold). Relative Importance (RI) represents the sum of Akaike weights across all relevant models (from Table S3) in which the predictor appeared. Variables with an RI ≥ 0.8 are considered strongly supported (Burnham et al., 2011; Symonds & Moussalli, 2011).

| Species | Variables | Estimate | SE | 95% CI | Relative Importance (RI) |
| --- | --- | --- | --- | --- | --- |
| *Cheirogaleus medius* | Forest humidity | 0.0376 | 0.0277 | [-0.0176, 0.0929] | 0.3522 |
|  | **Woody species richness** | **0.1452** | **0.0716** | **[0.0028, 0.2875]** | **0.8352** |
|  | Maximum temperature | 0.1135 | 0.1365 | [-0.1583, 0.3853] | 0.1298 |
|  | PC2 | -0.4016 | 0.2625 | [-0.9233, 0.1200] | 0.4508 |
|  | PC3 | -0.3487 | 0.3196 | [-0.9845, 0.2871] | 0.299 |
| *Microcebus ravelobensis* | **Forest humidity** | **0.0408** | **0.0195** | **[0.0021, 0.0795]** | **1.0000** |
|  | Woody species richness | 0.0435 | 0.0424 | [-0.0405, 0.1275] | 0.146 |
|  | Maximum temperature | -0.0516 | 0.0652 | [-0.1808, 0.0776] | 0.1175 |
|  | PC1 | 0.1696 | 0.1382 | [-0.1041, 0.4433] | 0.3080 |
|  | PC2 | -0.1894 | 0.1568 | [-0.4999, 0.1210] | 0.3051 |
|  | **PC3** | **-0.5714** | **0.1997** | **[-0.9668, -0.1760]** | **1.0000** |
| *Microcebus murinus* | Forest humidity | -0.0209 | 0.021 | [-0.0626, 0.0207] | 0.2249 |
|  | Woody species richness | 0.048 | 0.0416 | [-0.0345, 0.1304] | 0.1719 |
|  | Maximum temperature | -0.0875 | 0.0666 | [-0.2193, 0.0444] | 0.2246 |
|  | PC1 | 0.116 | 0.1454 | [-0.1719, 0.4040] | 0.1215 |
|  | PC2 | -0.0811 | 0.1429 | [-0.3642, 0.2020] | 0.104 |

**TABLE S9** Model-averaged parameter estimates predictors for lemur species richness. Estimates represent the effect size of each predictor on the response variable. SE (Standard Error) indicates the precision of the estimate. The 95% Confidence Interval (95% CI) shows the range of values within which the true effect is likely to fall. Predictors are considered influential if the confidence interval does not include zero (respective results in bold). Relative Importance (RI) represents the sum of Akaike weights across all relevant models (from Table S3) in which the predictor appeared. Variables with an RI ≥ 0.8 are considered strongly supported (Burnham et al., 2011; Symonds & Moussalli, 2011).

| Response | Predictor | Estimate | SE | 95% CI | Relative Importance (RI) |
| --- | --- | --- | --- | --- | --- |
| *Lemur species richness* | Forest humidity | 0.0048 | 0.0049 | [-0.0049, 0.0144] | 0.2249 |
|  | **Woody species richness** | **0.0433** | **0.0140** | **[0.0157, 0.0709]** | **1.0000** |
|  | Maximum temperature | 0.0092 | 0.0165 | [-0.0235, 0.0418] | 0.0823 |
|  | PC1 | 0.0646 | 0.0422 | [-0.0189, 0.1482] | 0.5883 |
|  | PC2 | -0.0339 | 0.0427 | [-0.1184, 0.0506] | 0.0965 |
|  | PC3 | -0.0337 | 0.0472 | [-0.1272, 0.0597] | 0.1798 |

**TABLE S10** Effects of fire history and severity on forest microclimate woody species richness, and vegetation structure (PCs 1-3). Two models (A, B) were fitted for each response variable based either on the entire dataset (model A) or only on the burnt partitions of the study sites (model B). The table reports estimates, standard errors (SE), 95% confidence intervals (CI) and the p-values with level of significance p < 5 for each predictor in all models.

| **Response Variable** | **Model** | **Predictor** | **Estimate** | **SE** | **95% CI** | **p-value** |
| --- | --- | --- | --- | --- | --- | --- |
| Forest humidity | A | Intercept | 4.36 | 0.044 | [ 4.28, 4.45] | < 0.0001*** |
|  |  | Burn status (Unburnt) | -0.00132 | 0.0242 | [-0.0487, 0.0460] | 0.984 |
|  |  | Number of fires | -0.000841 | 0.0151 | [-0.0304, 0.0287] | 0.964 |
|  | B | Intercept | 76.5 | 4.07 | [68.9, 84.4] | < 0.0001*** |
|  |  | Time since last fire | 0.170 | 0.100 | [-0.0261, 0.367] | 0.089 |
|  |  | Maximum fire severity | -2.22 | 13.6 | [-28.9, 24.4] | 0.871 |
| Woody species richness | A | Intercept | 13.7 | 1.15 | [11.4, 15.9] | < 0.0001*** |
|  |  | **Burn status (Unburnt)** | **5.05** | **1.16** | **[2.77, 7.32]** | < **0.0001***** |
|  |  | Number of fires | 0.032 | 0.5 | [-947, 1.01] | 0.948 |
|  | B | Intercept | 16.2 | 2.02 | [12.3, 20.2] | < 0.0001*** |
|  |  | Time since last fire | 0.054 | 0.068 | [-0.079, 0.188] | 0.428 |
|  |  | **Maximum fire severity** | **-33.1** | **8.25** | **[-49.2, -16.9]** | < **0.0001***** |
| PC1 | A | Intercept | 0.162 | 0.384 | [-0.591, 0.916] | 0.670 |
|  |  | **Burn status (Unburnt)** | **1.11** | **0.328** | **[0.464, 1.75]** | < **0.001**** |
|  |  | Number of fires | -0.254 | 0.135 | [-0.520, 0.0105] | 0.059 |
|  | B | Intercept | -0.343 | 0.387 | [-1.10, 0.415] | 0.376 |
|  |  | **Time since last fire** | **0.0510** | **0.0135** | **[0.0246, 0.0775]** | **0.0001***** |
|  |  | **Maximum fire severity** | **-10.5** | **2.62** | **[-15.6, -5.39]** | **< 0.0001***** |
| PC3 | A | Intercept | -0.0782 | 0.2610 | [-0.590, 0.433] | 0.765 |
|  |  | Burn status (Unburnt) | 0.0802 | 0.295 | [-0.498, 0.659] | 0.786 |
|  |  | Number of fires | 0.0379 | 0.113 | [-0.184,0.260] | 0.738 |
|  | B | Intercept | -0.665 | 0.394 | [-1.44, 0.107] | 0.091 |
|  |  | **Time since last fire** | **0.0244** | **0.0113** | **[0.00230, 0.0465]** | **0.030*** |
|  |  | Maximum fire severity | 3.02 | 2.13 | [-1.16, 7.20] | 0.157 |

**TABLE S11** Result of the Post-hoc Tukey test (R package emmeans, Lenth, 2023) used to compare the three forest zones (Burnt/Transition/Unburnt) regarding their forest humidity, woody species richness, PC1 and PC3*.* In bold: significant results.

| Models | Pairwise comparisons | Estimate | Standard error | Z-ratio | P-value |  |
| --- | --- | --- | --- | --- | --- | --- |
| Forest humidity | Burnt-Transition | 1.688 | 1.81 | 0.932 | 0.6211 |  |
|  | Burnt-Unburnt | 0.506 | 1.58 | 0.321 | 0.9447 |  |
|  | Transition-Unburnt | -1.181 | 2.32 | -0.510 | 0.8669 |  |
| Woody species richness | Burnt-Transition | 0.077 | 0.084 | 0.915 | 0.6305 |  |
|  | Burnt-Unburnt | -0.330 | 0.083 | -3.966 | **< 0.001**** |  |
|  | Transition-Unburnt | -0.408 | 0.110 | -3.705 | **< 0.001**** |  |
| PC1 | | Burnt-Transition | -0.212 | 0.327 | -0.649 | 0.7933 |
|  |  | Burnt-Unburnt | 1.134 | 0.342 | 3.311 | **< 0.01*** |
|  |  | Transition-Unburnt | 1.346 | 0.424 | 3.178 | **< 0.01*** |
| PC3 | | Burnt-Transition | -0.194 | 0.374 | -0.694 | 0.7676 |
|  |  | Burnt-Unburnt | 0.05 | 0.298 | 0.182 | 0.981 |
|  |  | Transition-Unburnt | 0.249 | 0.374 | 0.665 | 0.7840 |

**REFERENCES**

Burnham, K. P., Anderson, D. R., & Huyvaert, K. P. (2011). AIC model selection and multimodel inference in behavioral ecology: some background, observations, and comparisons. *Behavioral Ecology and Sociobiology*, *65*(1), 23–35. <https://doi.org/10.1007/s00265-010-1029-6>.

Lenth, R. (2023). emmeans: Estimated Marginal Means, aka Least-Squares Means_. R package version 1.8. 5.

Symonds, M. R. E., & Moussalli, A. (2011). A brief guide to model selection, multimodel inference and model averaging in behavioural ecology using Akaike’s information criterion. *Behavioral Ecology and Sociobiology*, 65(1), 13–21. https://doi.org/10.1007/s00265-010-1037-6.
